# Imaging-Based Screen Identifies Laminin 411 as a Physiologically Relevant Niche Factor with Importance for i-Hep Applications

**DOI:** 10.1016/j.stemcr.2018.01.025

**Published:** 2018-03-01

**Authors:** John Ong, Maria Paola Serra, Joe Segal, Ana-Maria Cujba, Soon Seng Ng, Richard Butler, Val Millar, Stephanie Hatch, Salman Zimri, Hiroyuki Koike, Karen Chan, Andrew Bonham, Michelle Walk, Ty Voss, Nigel Heaton, Ragai Mitry, Anil Dhawan, Daniel Ebner, Davide Danovi, Hiromitsu Nakauchi, S. Tamir Rashid

**Affiliations:** 1 Centre for Stem Cells and Regenerative Medicine & Institute for Liver Studies, King's College London, London SE1 9RT, UK; 2 The Gurdon Institute Imaging Facility, Cambridge University, Cambridge CB2 1QN, UK; 3 Target Discovery Institute, Oxford University, Oxford OX3 7FZ, UK; 4 Institute for Stem Cell Biology and Regenerative Medicine, Stanford University School of Medicine, Stanford, CA 94305, USA; 5 Perkin Elmer, Houston, TX 77055, USA

**Keywords:** iPS hepatocytes, extracellular niche, image-based screening, disease modeling, laminin

## Abstract

Use of hepatocytes derived from induced pluripotent stem cells (i-Heps) is limited by their functional differences in comparison with primary cells. Extracellular niche factors likely play a critical role in bridging this gap. Using image-based characterization (high content analysis; HCA) of freshly isolated hepatocytes from 17 human donors, we devised and validated an algorithm (Hepatocyte Likeness Index; HLI) for comparing the hepatic properties of cells against a physiological gold standard. The HLI was then applied in a targeted screen of extracellular niche factors to identify substrates driving i-Heps closer to the standard. Laminin 411, the top hit, was validated in two additional induced pluripotent stem cell (iPSC) lines, primary tissue, and an *in vitro* model of α1-antitrypsin deficiency. Cumulatively, these data provide a reference method to control and screen for i-Hep differentiation, identify Laminin 411 as a key niche protein, and underscore the importance of combining substrates, soluble factors, and HCA when developing iPSC applications.

## Highlights

• iPSC-derived hepatocytes (i-Heps) are functionally limited compared with primary cells
• Factors within the extracellular niche likely play a role in bridging this gap
• Laminin 411 was shown to be an important niche factor for i-Heps
• High content image analysis (HCA) can help development of i-Hep applications

## Teaser

Rashid and colleagues demonstrate the utility of a high-throughput imaging platform for identification of physiologically relevant extracellular niche factors to advance i-Heps closer to their primary tissue counterparts. The extracellular matrix (ECM) protein screen identified Laminin 411 as an important niche factor facilitating i-Hep-based disease modeling *in vitro*.

## Introduction

Stem cell-derived hepatocytes offer an exciting and novel resource for use in human disease modeling and cell therapy. Both embryonic (induced pluripotent stem cell [PSC]/embryonic stem cell [ESC]) and adult stem cell-derived products remain unsuitable for target downstream applications due to comparatively poor functionality when tested against primary adult counterparts (reviewed in Rashid et al., 2015). Extracellular signals delivered via a complex 3-dimensional protein-rich environment termed the “niche” tightly regulate hepatocyte function (Orford and Scadden, 2008, Morrison and Spradling, 2008). Recent attempts at enhancing the function of hepatocytes derived from iPSCs (i-Heps) by targeting the niche (Shan et al., 2013, Wang et al., 2017), though promising, are still unable to bridge the gap in functionality. One of the major reasons for this failure is the lack of a systematic approach to empirically define a signature of maturity that must be crossed in order for i-Heps to become suitably useful.

The aim of this study was therefore to (1) define a profile of healthy, freshly isolated primary hepatocytes (Hepatocyte Likeness Index; HLI) that cells of interest can be compared against for high-throughput screening (HTS), (2) apply this platform to screen hepatocyte niche factors for their ability to drive i-Heps closer to that target, and (3) validate our findings in a pharma-like screening environment.

## Results

### High Albumin Expression in Combination with Select Morphological Characteristics Defines a Functionally Relevant Hepatocyte Signature and Enables Calculation of the Hepatocyte Likeness Index

**Figure 1:**
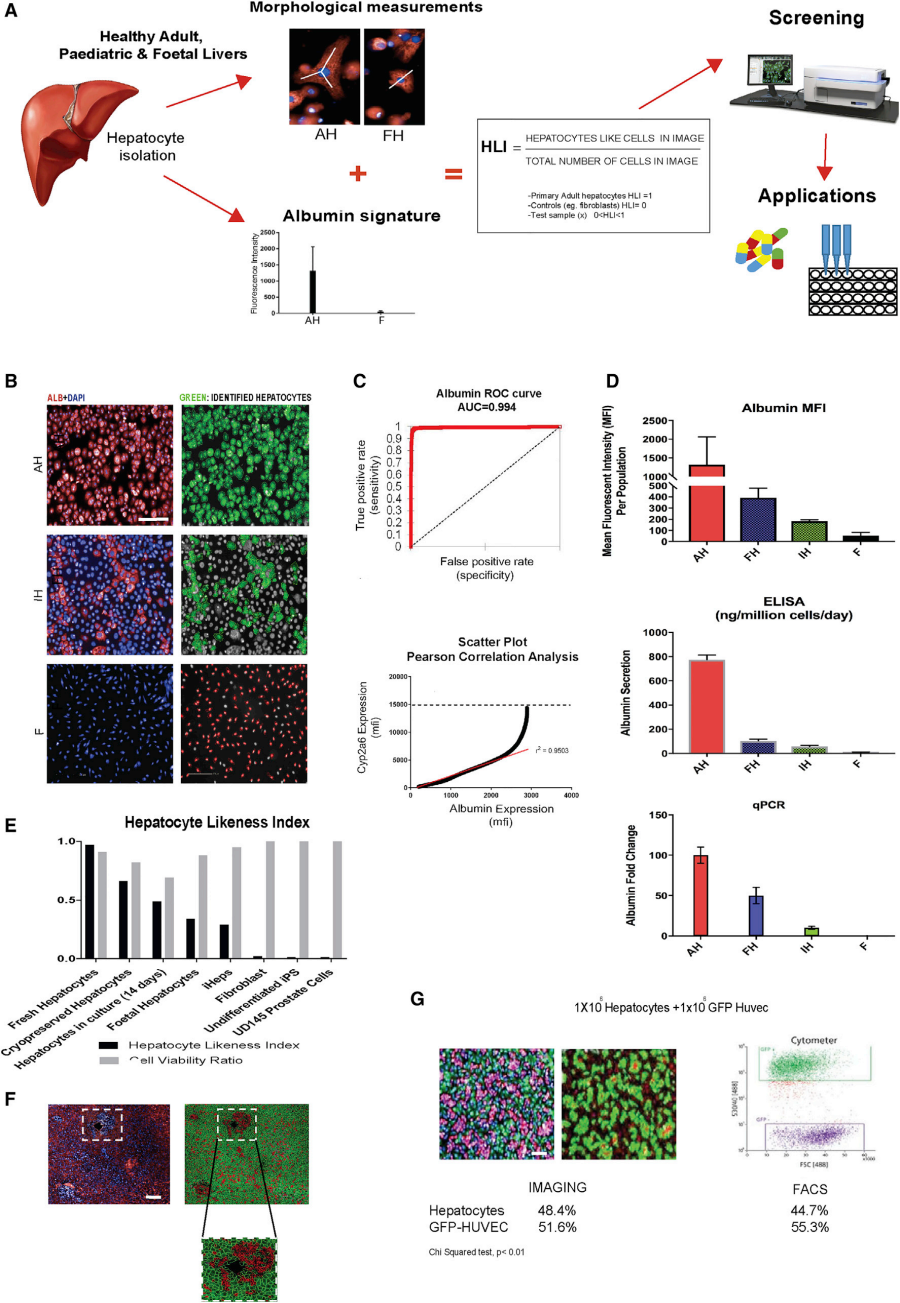
Generation of the Hepatocyte Likeness Index (A) Schematic representation of work-flow that established morphological parameters and protein signatures for the Hepatocyte Likeness Index (HLI) and its validation across different cell types. AH, adult hepatocytes (n = 17 donors); F, fibroblasts (n = 3 donors). (B) Image-based algorithm enables the automated identification of hepatocytes by morphology and albumin. (Left column) DAPI and albumin staining in adult hepatocytes (AH), i-Heps (IH), and fibroblasts (F). (Right column) Cells highlighted green represent identified hepatocytes; gray or red highlighted cells are recognized as non-hepatocytes. Scale bar, 200 μm. (C) (Top) Receiver-operating characteristic curve demonstrating high diagnostic ability of albumin as a surrogate marker of hepatocyte likeness and maturity. (Bottom) Pearson correlation analysis of showing that high albumin expression is closely related with metabolic (cytochrome 2A6) function (Pearson coefficient r^2^ = 0.9503, 95% confidence interval, p < 0.01). (D) Mean fluorescence intensity (MFI) of albumin is validated by ELISA and qPCR in adult hepatocytes (AH), fetal hepatocytes (FH), i-Heps (IH), and fibroblasts (F). n = 9 donors (AH), n = 4 donors (FH), n = 3 donors (IH), and n = 3 donors (F). Error bars show mean ± SD. (E) HLI discriminates freshly isolated adult hepatocytes from cryopreserved adult, cultured adult, freshly isolated fetal hepatocytes, and i-Heps in a manner independent of cell viability (n = 3 different biological samples in all groups, all independent experiments). (F) HLI discriminates hepatocytes from other cells *in vivo*. (Left panel) Human liver section (10×) stained for albumin (red) demonstrating albumin-positive and albumin-negative (blue) cells. (Right panel) Application of HLI algorithm enables automated detection of hepatocytes (green) from non-hepatocytes (red) by machine learning. Enlarged square: accurate distribution of hepatocytes and non-hepatocyte cells around periportal area. Scale bar, 100 μm. (G) HLI discriminates hepatocytes from other cells *in vitro*. Co-culture of GFP-labeled HUVECs (green) and hepatocytes (red) (left panel) were discriminated by HLI (right panel) better than by flow cytometry (right). Scale bar, 100 μm.

To define the profile of a target healthy hepatocyte, we first measured hepatocyte morphology and protein expression in hepatocytes freshly isolated from normal fetal, neonatal, and adult livers (n = 17, Table S1) and used this information to construct the HLI for comparing other cell types of interest (Figure 1).

Measurement of morphological properties revealed statistically significant differences in all parameters between fetal, pediatric, and adult hepatocytes (Figure S1). True multivariate analysis (Random Forest) identified cell width, cell area, and cell symmetry as the three most important characteristics that differentiated human hepatocytes from non-hepatocyte cell types. Compared with adult hepatocytes, fetal hepatocytes were smaller and less symmetrical. Nuclear area and width also distinguished the physiological age of primary hepatocytes with nuclear polyploidy, as expected, being observed in approximately 30% of hepatocytes from adult liver samples but in less than 5% of cells from fetal liver. i-Heps, on the other hand, resembled primary adult hepatocytes in symmetry but had larger nuclei. Significant variation in their nuclear and cell sizes was also observed, with larger i-Hep morphology correlating poorly with albumin expression. Though promising, the discriminatory ability offered by the above morphological analyses was therefore not enough to proceed to HTS. To improve the accuracy of our index, we therefore evaluated the added value of simultaneously measuring protein expression. Quantification of protein expression by fluorescence intensity was previously widely reported for a broad spectrum of biological applications similar to ours (Young et al., 2008, Massey, 2015, Boehnke et al., 2016, Rodriguez-Muela et al., 2017), making the approach suitable for this study. We therefore tested in different concentrations, exposure times, and excitation levels a panel of immunofluorescent antibodies whose target proteins had previously been reported to correlate with hepatocyte function (Rowe et al., 2013, Baxter et al., 2015) (Figure S2). Analysis of protein expression by mean fluorescence intensity (MFI) in this manner showed discriminatory signatures between i-Heps, fetal hepatocytes, and adult hepatocytes. Albumin expression was found to be the most accurate biomarker for hepatocyte identification in this regard (Figure 1B), correlating best with cellular metabolic function (Figure 1C). The validity of albumin MFI as a surrogate marker for cellular albumin protein level was then confirmed using orthogonal assays such as qPCR and ELISA (Figure 1D). Reassuringly, these experiments demonstrated that increased albumin MFI levels corresponded with relative increases in hepatic function across multiple independent experiments and biological samples.

**Figure 2:**
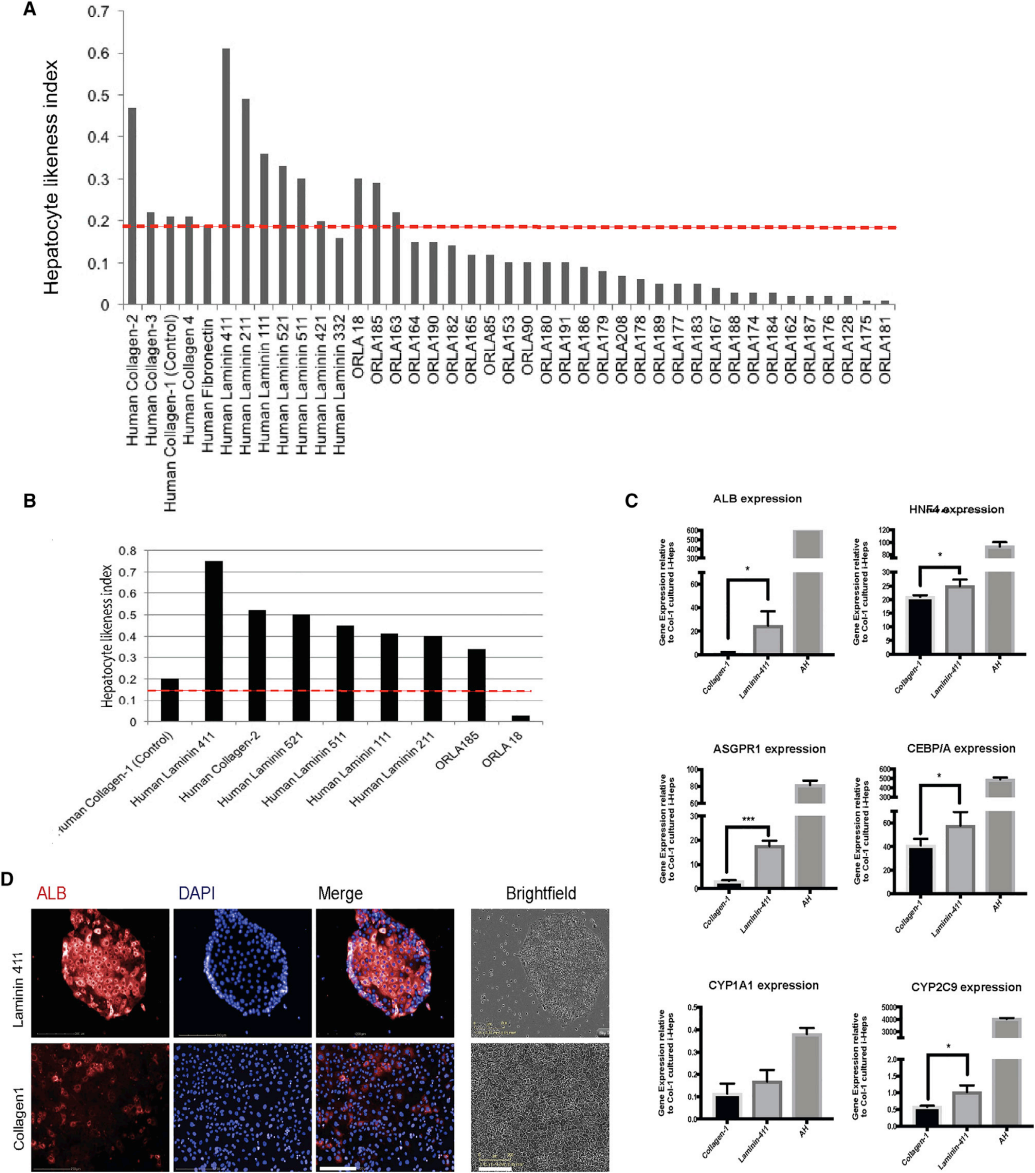
Screening of Niche Factors Using HLI Algorithm Demonstrates Effect of Laminin 411 in i-Heps (A) The HLI algorithm (y axis) was used to screen for the effects of 58 different hepatocyte niche factors (x axis) on i-Heps 7 days after plating. Control line (red) is the control threshold based on culture with collagen-1. (B) Revalidation of the eight hits from the first round of screening. (C) Relative gene expression of i-Heps cultured on Laminin 411 (middle bars) compared with collagen-1 (left bars) with expression levels in freshly isolated adult hepatocytes (AH) as control (right bars). n = 3 (different i-Hep cell lines and independent experiments); error bars show mean ± SD; ^∗^p < 0.05, ^∗∗∗^p < 0.01. (D) Immunofluorescence staining for albumin (red, left), DAPI (blue, left middle) and merge (middle right), of i-Heps cultured on Laminin 411 (top) versus collagen-1 (bottom); cell morphology is shown on far right (10× magnification; scale bar, 100 μm). Images shown represent n = 3 different biological replicates and independent experiments. Scale bar, 200 μm.

Incorporating the above data (morphology + albumin MFI), an analysis sequence that enabled supervised machine learning was constructed for the automated identification of bona fide hepatocytes—firstly by nuclear morphology, then cell morphology, and finally albumin expression (Figure S3). Cells satisfying normal parameters (of freshly isolated hepatocytes) were classified as “hepatocyte positive” and others “hepatocyte negative.” The HLI was then defined as the proportion of positively identified hepatocytes over the total number of cells analyzed. Therefore, in a population of healthy primary adult hepatocytes HLI approximates 1 and in a negative biological control (e.g. fibroblasts), HLI approximates 0. We next validated the physiological relevance of this index by measuring HLI in different cell populations (Figure 1E) to show how the algorithm could be used to not only differentiate hepatocytes from non-hepatic cells but also to discriminate freshly isolated adult hepatocytes from cryopreserved adult, cultured adult, and freshly isolated fetal hepatocytes in a manner independent of cell viability (Figure 1E). The HLI was also capable of identifying bona fide hepatocytes within heterogeneous cell populations such as is found in human liver tissue (Figure 1F) or in *in vitro* co-culture systems (Figure 1G) with a higher accuracy than flow cytometry. Finally, we demonstrated that the HLI could readily be transferred to other image analysis platforms (Figure S3) before proceeding to screen for the effects of hepatocyte niche factors on i-Hep HLI (Figure 2).

### A Screen of ECM Proteins and Soluble Niche Factors Demonstrates that Laminin 411 Advances i-Heps toward Functional Significance

Using the Human Matrisome Project database (http://matrisomeproject.mit.edu) we identified 105 proteins likely to be important in hepatocyte maturation. Of these, a total of 58 proteins and niche factors were put forward into the screen based on biological interest and commercial availability (Table S2). From the initial screen, eight proteins (Figure 2A) were found to have a positive effect (HLI > 0.2) and taken forward for validation. Seven of the eight proteins were found to be efficacious in the second round (Figure 2B). The hit with the greatest effect on HLI, Laminin 411, was then tested on i-Heps from three different biological samples. In these conditions, cells displayed higher expression levels of genes known to be associated with adult hepatocyte function such as *CYP2C9*, *ASGPR1*, *HNF4A*, *CEBP/A*, and *ALB* (Figure 2C). Finally, immunofluorescence staining for albumin confirmed that i-Heps cultured in Laminin 411 for 2 weeks have higher protein expression with more cells meeting morphological parameters of a normal hepatocyte (Figure 2D).

### Laminin 411 Is a Component of the Hepatic Niche in Human Fetal Liver

**Figure 3:**
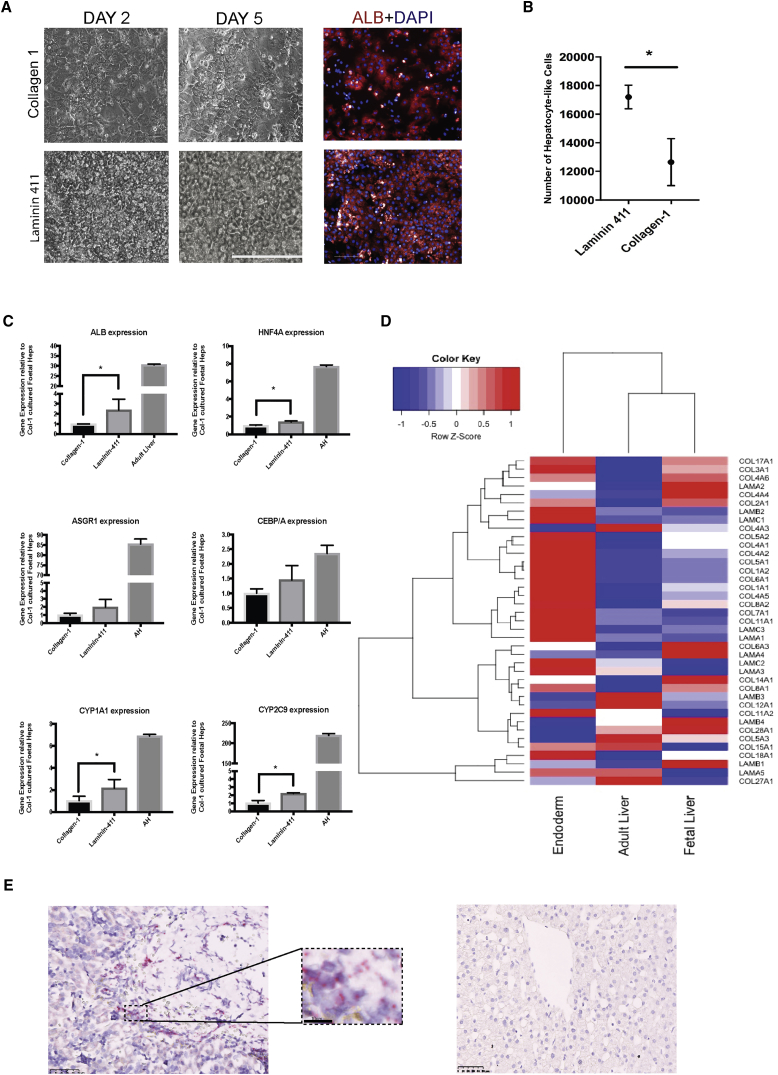
Laminin 411 Is a Physiologically Relevant Niche Factor in Fetal Liver Development (A) Morphology (20×) of fetal hepatocytes cultured on collagen-1 (top) versus Laminin 411 (bottom) at 2 (left) and 5 (middle) days post plating (scale bar, 200 μm). Immunofluorescence staining (right) for albumin (red) plus DAPI (blue) at 5 days post plating (scale bar, 100 μm). (B) Number of albumin-expressing fetal hepatocytes identified by HLI algorithm (y axis), cultured on Laminin 411 versus collagen (x axis) at day 5. n = 3 different biological samples and independent experiments; data presented as mean ± SD; ^∗^p < 0.05. (C) Relative gene expression (y axis) of fetal hepatocytes cultured for 7 days on Laminin 411 (middle bars) versus collagen 1 (left bars) compared with adult liver (right bars). n = 3 (different biological samples and independent experiments); data presented as mean ± SD; ^∗^p < 0.05. (D) ECM gene expression heatmap comparing iPSC-derived endoderm (left) with adult (middle) and fetal (right) liver. (E) RNA *in situ* hybridization for *LAMA4* on a 16-pcw liver slide (left) (40× magnification; scale bar, 50 μm). Detail of the red dots expanded in the square compared with adult liver (right).

Next, we investigated the importance of Laminin 411 during human liver development. We obtained freshly isolated human fetal hepatocytes from 16- to 20-pcw (post-coital weeks) donor tissue (n = 3 donors) and observed similar effects of culturing these cells on Laminin 411 as with i-Heps. Compared with collagen-1, Laminin 411 improved cell survival and morphology (Figure 3A) while retaining a higher population of cells mirroring the adult hepatocyte phenotype (Figure 3B). Gene expression analysis confirmed a statistically significant increase in the expression of *ALB*, *HNF4A*, *CYPA1*, and *CYP2C9* (Figure 3C). We then hypothesized that if Laminin 411 is relevant to human physiology of hepatocytes, it would also be expressed in liver. For this purpose, we analyzed gene expression databases for genes expressing ECM proteins in adult versus fetal versus iPSC-endoderm tissue. This analysis demonstrated upregulation of *LAMA4* and *LAMB1* (the constituent components of LAM-411) in human fetal liver (Figure 3D). We confirmed this computational presumption using RNA *in situ* hybridization, and found high expression of *LAMA4* near vascularized regions of maturing human fetal liver and only very weak expression in adult liver (Figure 3E).

### Laminin 411 Prolonged Survival of Hepatic Progenitor Cells and Is Better Suited for i-Hep-Based Drug-Screening Applications

**Figure 4:**
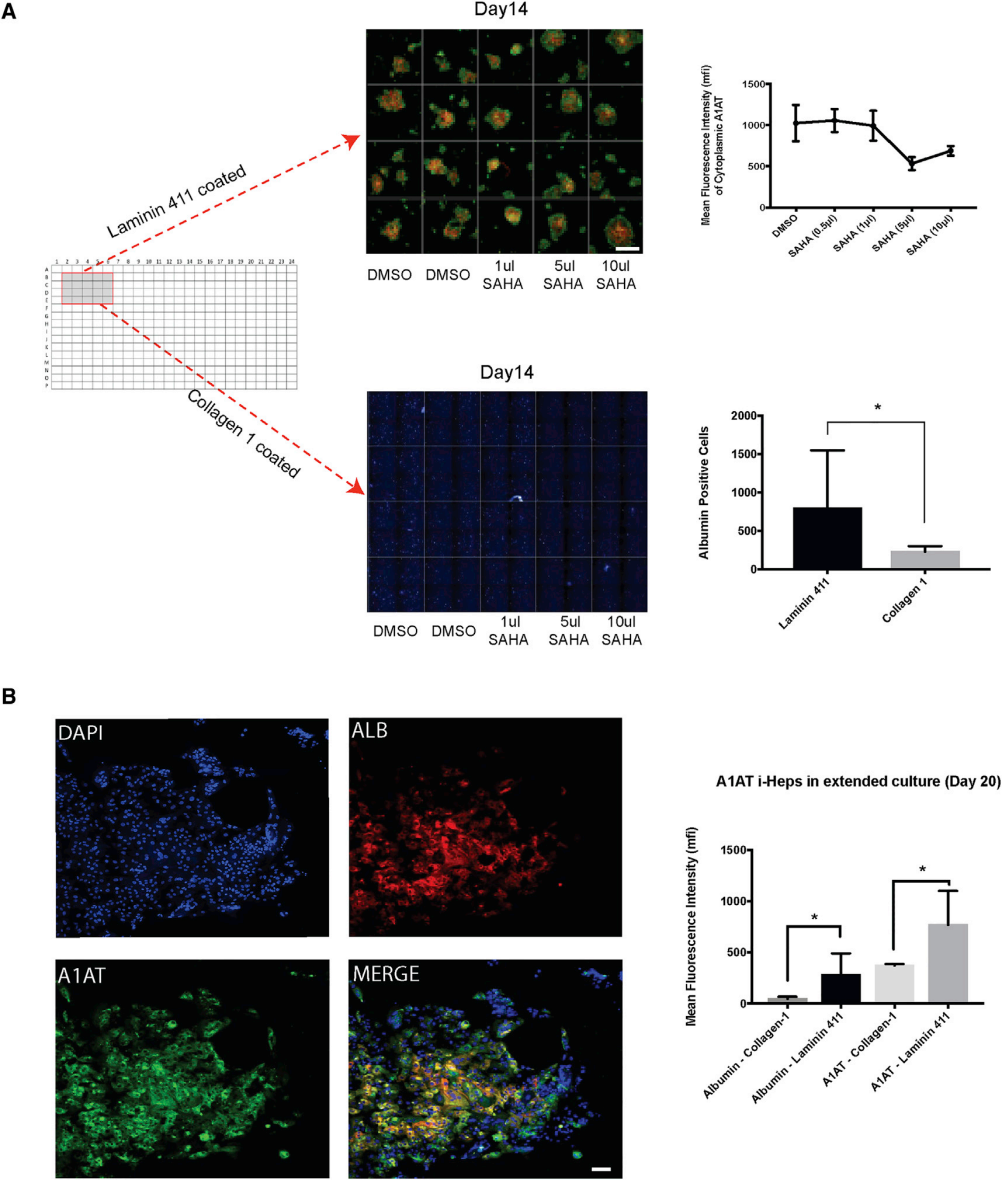
Laminin 411 Is Better Suited for Drug Screening in an i-Hep Disease Model of α1-Antitrypsin Deficiency (A) I-Heps plated on 384-well plates coated with Laminin 411 or collagen-1 (left) were cultured for 14 days then imaged using the HLI (scale bar, 1 mm) i-Heps cultured on Laminin 411 responded to SAHA treatment (top right) and have more function than albumin-producing cells (bottom right). Data presented as mean ± SD; n = 3 biological replicates from independent experiments; ^∗^p < 0.05. (B) Enlarged images from (A) showing immunofluorescence staining of i-Heps, DAPI (blue top left), albumin (red top right), A1AT (green bottom left), and MERGE (yellow bottom right; scale bar, 50 μm). MFI for images were quantified (right graph) and showed higher MFI of both A1AT and albumin in Laminin 411 (middle bars) compared with collagen-1 (outer bars). Data presented as mean ± SD; n = 3 biological replicates from independent experiments; ^∗^p < 0.05.

Lastly we sought to examine whether our findings had identified a substrate more suitable for use with an iPSC α1-antitrypsin deficiency (A1AT-Z) disease model drug-screening platform (Yusa et al., 2011). Cells were seeded into 384-well plates coated with collagen-1 or Laminin 411 and evaluated for survival, disease signature, inter-well variability, and response to the histone deacetylase (HDAC) inhibitor suberoylanilide hydroxamic acid (SAHA) (Bouchecareilh et al., 2012). After 28 days in culture (Figure 4A), Laminin 411 demonstrated improved cell survival and had a stronger disease signature of A1AT (Figure 4B) compared with collagen-1 when stained for albumin and A1AT protein. Improvement in cell survival was also observed in primary fetal hepatocytes; however, similarly to current literature this effect was not seen in primary adult hepatocytes (Watanabe et al., 2016).

## Discussion

Here, we report the development of an imaging-based algorithm capable of stratifying human hepatocyte maturity in a high-throughput manner. With this capability, candidate extracellular niche factors were screened for their capacity to drive i-Heps closer to hepatocytes freshly isolated from human liver. The efficacy of the most significant hit, Laminin 411, was confirmed in three different iPSC lines and three different human fetal hepatocyte samples. Translational relevance was then demonstrated by superior performance of the substrate in a high-throughput drug-screening platform for α1-antitrypsin deficiency.

Imaging-based assays represent an excellent modality for studying the effects of extracellular niche components in a high-throughput manner. Bhatia and colleagues recently reported the development of an exciting high-throughput, imaging-based genetic screening platform to identify the most critical stromal cell gene products involved in stabilization of primary human hepatocyte functions (Shan et al., 2016). Our work extends the significance of such studies by using freshly isolated hepatocytes, the physiological gold standard in the field (instead of frozen cells) to construct a “hepatocyte-ness” score (the HLI) for measuring the effects of substrates on stem cell-derived hepatocytes. These tools are, we believe, essential for expediting effective translational outputs in this field, with the generalizability of the platform (reproducibility across multiple image analysis platforms, cell lines, and commercially available substrates) suggesting that rapid adaptation by others could easily be achieved.

Results from the screen performed highlighted the important role played by laminins, a group of heterotrimeric ECM proteins, in the hepatic progenitor response. This association had previously been reported in adult liver (Kallis et al., 2011) and in cholangiocyte differentiation (Takayama et al., 2016) but not in embryonic hepatocyte development. Here we demonstrated Laminin 411 to be a biologically relevant and important factor in advancing i-Hep differentiation and in human hepatic fetal development (Figure 3). Although its specific role remains unclear, its structure comprising α-4 (LAMA4), β-1, and γ-1 chains, facilitates binding to α_6_β_1_ and α_6_β_4_ integrin receptors, suggesting that its effects may be due to activation of several receptors or pathways yet to be fully elucidated. This heterogeneity opens up the likelihood of downstream function being a consequence of co-engagement with a combination of as yet poorly defined soluble and insoluble factors. Our future efforts will therefore seek to unravel these complex interactions by combining soluble factors with insoluble ECM proteins in more sophisticated HTS screens. In the meantime, as shown by our A1AT drug-screening data (Figure 4), information from even the most basic of screens using the algorithm can rapidly be translated into meaningful advances, which in turn suggests that our approach could be of widespread utility in the field.

## Experimental Procedures

### Cells

All human tissues were collected with informed consent following ethical and institutional guidelines. Freshly isolated hepatocytes were obtained from Triangle Research Labs, the Institute of Liver Studies (King's College Hospital), while fetal livers were obtained from the Human Developmental Biology Resource of University College London. Isolated adult hepatocytes were plated on 96-well collagen-1-coated plates (Greiner) at a density of 10,000 cells per well and maintained in commercially supplied media. Fetal hepatocytes were obtained from 16- to 20-week-old fetuses, dissociated as previously described (Gramignoli et al., 2012) and cultured in collagen-1 or Laminin 411 (Biolamina, 10 μg/mL) while being maintained on DMEM supplemented with insulin (Sigma) and dexamethasone (Sigma) (10^−7^ M).

Human i-Heps were obtained from Definigen as a cryopreserved sample and recovered for 10 days as per manufacturer’s instructions (ref. 16; A, patient 2 line 1; B, patient 1 line 1; C, patient 3 line 1) or supplied from the Nakauchi lab (TkDA line as used in Takebe et al., 2013). Cells were plated on flat-bottomed 96-well plates pre-coated with collagen-1 or Laminin 411 (as previously described) at a density of 1.25 × 10^5^ cells per well. Cultures were maintained in Hepatozyme medium (Life Technologies), supplemented with 1% L-glutamine, 1% penicillin/streptomycin, 2% non-essential amino acids (Life Technologies), 2% lipid concentrate (Life Technologies), 0.1% insulin-transferrin-selenium (Sigma), 0.01 μg/mL oncostatin M (Peprotech), and 0.05 μg/mL hepatocyte growth factor (Peprotech).

For the co-culture system, hepatocytes were cultured in a 1:1 ratio with nuclear GFP-labeled human umbilical vein endothelial cells (HUVECs) (Essen Bioscience) using a 1:1 mix of Hepatozyme and EGM (Lonza).

Human fibroblasts from three donors were isolated from skin explants dissociation and cultured in collagen-coated 96-well plates at a density of 500,000 cells/mL.

Cell viability was determined by trypan blue (Invitrogen) exclusion and all cells were fixed 4% (w/v) paraformaldehyde phosphate buffer solution (PFA; Sigma) after 48 hr of plating.

### ECM Screening

Proteins were used individually, in pairs, or in combinations of three at five different concentrations by overnight coating of 96-well plates. Concentrations used for coating were dependent on the target protein. I-Heps were plated for 7, 14, and 28 days before being fixed, stained, and imaged as per the protocol defined earlier. The most significant hits from the first round were taken forward into a secondary screen using two additional iPSC cell lines generated using two separate protocols (Rashid et al., 2010, Mallanna and Duncan, 2013).

### Image Analysis

Staining with CellMask blue was used for cell segmentation, images captured with Operetta (PerkinElmer), and analysis performed by PhenoLOGIC software (PerkinElmer) to identify parameters that co-relate most closely with physiological hepatocyte likeness. Albumin staining in primary hepatocytes was found to be equal to CellMask blue for cell segmentation. Areas under receiver-operating characteristic curves were plotted using XLSTAT and Prism (GraphPad) to determine the optimum cutoff fluorescent intensities.

For the 384-well plates, images were taken with a 10× objective on the GE IN Cell Analyzer 6000.

### Immunofluorescent Staining

After fixation, cells were blocked and permeabilized in 1% (w/v) BSA (Sigma-Aldrich), 3% donkey serum (Life Technologies), and 0.1% Triton X-100. Primary antibodies (Table S3) were applied for 1 hr. After washes, cells were incubated with Alexa 647, Alexa 568, Jackson 488-conjugated secondary antibodies (Life Technologies). Slides were counterstained with DAPI (NucBlue) (Life Technologies).

Paraffin-embedded sections (5 μm thickness) were dewaxed, rehydrated, and subjected to an antigen retrieval procedure with sodium citrate (Sigma) (pH 6.0) before following the staining procedure as described above.

### Real-Time PCR Analysis

Total RNA was harvested using TRI reagent (Sigma), treated with DNase (Promega), and phenol/chloroform purified. For each sample 0.5 μg of total RNA was reverse transcribed using a SuperScript VILO cDNA Synthesis kit (Thermo Fisher Scientific). A typical RT-PCR reaction contained 10 ng of sample cDNA, 0.0075 μL of individual forward and reverse primer each at 100 μM stock, 5 μL of Taqman Universal Master mix (Applied Biosystems), 1 μL of Taqman target probe (Table S4), and made up to 10 μL with nuclease-free water. Real-time PCR reactions were amplified for 40 cycles on a CFX384 Touch Real-Time PCR Detection System (Bio-Rad) in triplicate and normalized to ACTB (Figure S4) in the same run. Real-time RT-PCR data are presented as the mean of three independent biological experiments, and error bars indicate SEM.

### Heatmap Generation

Heatmaps were generated from bulk RNA-sequencing data collected from three human fetal livers (two 14-pcw samples and one 16 pcw), three human adult livers (female 18 years, male 13 years, and female 36 years), and three i-Hep samples harvested at day 6 (endodermal stage) from the BOB cell line. Starting with 1 μg input total RNA, rRNA was removed using a Ribo-Zero Gold rRNA Removal kit (Illumina). Sequencing libraries were prepared using NEBNext Ultra Directional RNA Library Prep Kit for Illumina (NEB) using 100 ng of rRNA-depleted sample and sequenced on a HiSeq 2500 system in rapid run mode (Illumina) following a standard protocol. All libraries generated between 15 and 25 million reads. Reads were mapped to GRCh38 reference genome using Bowtie2 (Langmead and Salzberg, 2012). Raw counts and normalized gene expression were generated using HT-Seq (Anders et al., 2015) and DESEq2 (Love et al., 2014) packages, respectively. The heatmap was generated using R (http://www.R-project.org/) (R Development Core Team, 2008). Heatmaps represent average DESEq2 normalized gene expression values of three independent biological samples.

### Human Albumin ELISA

Albumin production of all different cell types was assessed using the Human Albumin Quantification Set (Bethyl Laboratories) following the manufacturer's instructions. Results shown are the mean and SD of triplicate biological replicates. Absorbance was measured using a GloMax-Multi Detection System (Promega) at 450 nm.

### *In Situ* Hybridization

*In situ* hybridization for *LAMA4* (Hs-LAMA4, cat. No. 459161) expression was performed on 5-μm sections of 15- to 17-pcw fetal and adult livers using the RNAscope 2.5 High Definition (Red, cat. no. 322350, Advanced Cell Diagnostics, Hayward, CA) according to the manufacturer’s instructions. Slides were counterstained with Gill's hematoxylin and analyzed under the Hamamatsu scanner.

### Data Analysis

Data were analyzed with GraphPad Prism for Pearson Correlation Analyses and t tests. t Tests were plotted as mean ± SD or mean ± SEM where specified. Phenologic and Spotfire (PerkinElmer) were also used for multivariate analysis (Random Forrest), PCA plot, and SOM algorithm.

## Author Contributions

S.T.R., H.N., and J.O. designed the experiments, which were carried out by J.S., A.-M.C., S.S.N., R.B., V.M., S.H., S.Z., H.K., K.C., A.B., M.W., T.V., N.H., R.M., A.D., D.E., and D.D., led by J.O. and M.P.S. All authors contributed to data analysis and writing of the manuscript, led by J.O., M.P.S., H.N., and S.T.R.

## Supplemental Information

Document S1. Figures S1–S4 and Tables S1–S4

Document S2. Article plus Supplemental Information
