# Supplementary material for: Imaging-Based Screen Identifies Laminin 411 as a Physiologically Relevant Niche Factor with Importance for i-Hep Applications
Source: Stem Cell Reports. 2018 Mar 1;10(3):693–702. doi: 10.1016/j.stemcr.2018.01.025 (PMC5919292; doi:10.1016/j.stemcr.2018.01.025)
Supplement: Document S1. Figures S1–S4 and Tables S1–S4 [file mmc1.pdf]

Stem Cell Reports, Volume *10*

Supplemental Information

Imaging-Based Screen Identifies Laminin 411 as a Physiologically Relevant Niche Factor with Importance for i-Hep Applications

John Ong, Maria Paola Serra, Joe Segal, Ana-Maria Cujba, Soon Seng Ng, Richard Butler, Val Millar, Stephanie Hatch, Salman Zimri, Hiroyuki Koike, Karen Chan, Andrew Bonham, Michelle Walk, Ty Voss, Nigel Heaton, Ragai Mitry, Anil Dhawan, Daniel Ebner, Davide Danovi, Hiromitsu Nakauchi, and S. Tamir Rashid

Table S1. Details of hepatocyte donors, i-Hep cell lines and negative controls, related to text in Results.

Adult Livers - History of liver disease, Hepatitis B, Hepatitis C and HIV excluded				
Age of Donor	Race	Gender	Cell Viability	Comments
21 years old	Caucasian	Male	90%	Tobacco: Yes<br>Alcohol: No<br>BMI 30
21 years old	Caucasian	Male	88%	Tobacco: Yes<br>Alcohol: 2 units/week<br>BMI 24, Heroin use
29 years old	Caucasian	Male	93%	Tobacco use: No<br>Alcohol: No<br>BMI 25.5
30 years old	African-American	Female	89%	Tobacco: No<br>Alcohol: No<br>BMI 33.2
34 years old	Caucasian	Female	95%	Tobacco use: Yes<br>Alcohol: No<br>BMI 26
39 years old	Caucasian	Male	92%	Tobacco: Yes<br>Alcohol: 2 units/week<br>BMI 21, Heroin use
46 years old	Caucasian	Female	90%	Tobacco: No<br>Alcohol: No<br>BMI 24.0
48 years old	Hispanic	Female	90%	Tobacco: No<br>Alcohol: No<br>BMI 25.4
30 years old	African-American	Male	91%	Tobacco: No<br>Alcohol: No<br>BMI: 20.3
**Paediatric Livers - History of liver disease, Hepatitis B, Hepatitis C and HIV excluded**				
Age of Donor	Race	Gender	Cell Viability	Comments
1 month old	Hispanic	Male	83%	Unknown
14 month old	Caucasian	Female	94%	BMI 20.7
20 months	Caucasian	Female	90%	BMI 21
11 years old	Caucasian	Male	86%	Unknown
**Fetal Livers (Maternal serology negative for Hepatitis B, Hepatitis C and HIV)**				
Age of Donor	Race	Gender	Cell Viability	Comments
19 weeks	Caucasian	Unknown	78%	Termination of pregnancy
21 weeks	Caucasian	Unknown	82%	Termination of pregnancy
20 weeks	Caucasian	Unknown	80%	Termination of pregnancy
17 weeks	Caucasian	Unknown	85%	Termination of pregnancy

Negative Controls			
Sample	Age of Donor	Race, Gender	Comments
Fibroblast 1	51 years old	Caucasian, female	Isolated from skin
Fibroblast 2	44 years old	Caucasian, female	Isolated from skin
Fibroblast 3	40 years old	Caucasian	Isolated from foreskin of adult male
iPS RMA	50years old	Male	Undifferentiated iPS cell line
IPS HDB	Unknown	Unknown	Undifferentiated iPS cell line
DU145	Unknown	Unknown	Prostate Cancer Cell Line

**iPS derived Hepatocytes (i-Heps)**	
i-Hep 1	Wild-type (Rashid et al JCI 2010)
i-Hep 2	Alpha-1 antitrypsin deficiency (Rashid et al JCI 2010)
i-Hep 3	Wild-type (TkDA Takebe et al Nature 2013)

Table S2. List of ECM / Niche Factors Tested, related to text in Results section.

S/N	Protein / Peptide	Source, Catalogue number
1	Human Collagen-1	Abcam Ab7533
2	Human Collagen-2	Abcam Ab 7534
3	Human Collagen-3	Abcam Ab 7535
4	Human Collagen-4	Abcam Ab7536
5	Human Fibronectin	Merck Millipore FC010
6	Human Laminin 111	Biolamina, Lamscreen KT202
7	Human Laminin 211	Biolamina, Lamscreen KT202
8	Human Laminin 332	Biolamina, Lamscreen KT202
9	Human Laminin 411	Biolamina, Lamscreen KT202
10	Human Laminin 421	Biolamina, Lamscreen KT202
11	Human Laminin 511	Biolamina, Lamscreen KT202
12	Human Laminin 521	Biolamina, Lamscreen KT202
13	Human CYR61	R&D systems, 4055-CR-050
14	Human LRRCC17	Abcam, Ab 160894
15	Human Vitronectin	R&D systems, 2308-VN-050
16	Human Wnt3a	R&D systems, 5036-WN-010
17	Human Wnt5a	R&D systems, 645-WN-010
18	Human Wnt 5b	R&D systems, 7347-WN-025
19	Human Wnt7a	R&D systems, 3008-WN-025
20	Mouse Wnt9a	R&D systems, 8148-WN-025
21	Mouse Wnt9b	Stanford University, Nusse Lab
22	Human EGFL7	Abcam, Ab 218567
23	Human Interleukin 3	R&D systems, 203-IL-010
24	Human Interleukin 4	R&D systems, 6507-IL-010
25	Human Interleukin 6	R&D systems, 7270-IL-025
26	Adrenaline	Sigma Aldrich, Y0000882
27	Serotonin	Sigma Aldrich, H9523
28	Levothyroxine	Sigma Aldrich, L0570000
29	Dexamethasone	Sigma Aldrich, D4902-25MG
30	Insulin	Sigma Aldrich, I3536
31	Ascorbic acid	Sigma Aldrich, PHR1008-2G
32	Protein ORLA 153 (RGDS sequence)	ORLA Proteins, ORLA 153
33	Protein ORLA 164 (PHSRN sequence)	ORLA Proteins ORLA 164
34	Protein ORLA 208 (CS3/Variable domain)	ORLA Proteins ORLA 208
35	Protein ORLA 162 (IKVAV sequence)	ORLA Proteins ORLA 162
36	Protein ORLA 187 (VQLRNGFPYFSY sequence)	ORLA Proteins ORLA 187
37	Protein ORLA 188 (GLLFYMARINHA sequence)	ORLA Proteins ORLA 188
38	Protein ORLA 189 (IKVSV sequence)	ORLA Proteins ORLA 189
39	Protein ORLA 163 (YIGSR sequence)	ORLA Proteins ORLA 163
40	Protein ORLA 165 (MNYYSNS sequence)	ORLA Proteins ORLA 165
41	Protein ORLA 185 (GTPGPQGIAGQRW sequence)	ORLA Proteins ORLA 185
42	Protein ORLA 186 (VEGF mimetic)	ORLA Proteins ORLA 186
43	Protein ORLA 175 (IPKASSVPTELSAISMLYYLDENEKVVLK)	ORLA Proteins ORLA 175
44	Protein ORLA 176 (PQVTRGDVFTM sequence)	ORLA Proteins ORLA 176
45	Protein ORLA 177 (KKQRFRHRNRKGYRSQ sequence)	ORLA Proteins ORLA 177
46	Protein ORLA 178 (VDTYDGRGDSVVYGLRSKSK sequence)	ORLA Proteins ORLA 178
47	Protein ORLA 174 (FHRRIKA Heparin binding)	ORLA Proteins ORLA 174
48	Protein ORLA 179 (VFDNFVLK sequence)	ORLA Proteins ORLA 179
49	Protein ORLA 90 (Fibroblast Growth Factor 1 - acdic)	ORLA Proteins ORLA 90
50	Protein ORLA 128 (Fibroblast Growth Factor 2 - basic)	ORLA Proteins ORLA 128
51	Protein ORLA 181 (Fibroblast Growth Factor 4)	ORLA Proteins ORLA 181
52	Protein ORLA 183 (Stem Cell Factor soluble kit ligand)	ORLA Proteins ORLA 183
53	Protein ORLA 184 (Sonic hedgehog)	ORLA Proteins ORLA 184
54	Protein ORLA 167 (Epidermal Growth Factor)	ORLA Proteins ORLA 167
55	Protein ORLA 180 (Leukaemia Inhibitory Factor)	ORLA Proteins ORLA 180
56	Protein ORLA 190 (GM-Colony Stimulating Factor)	ORLA Proteins ORLA 190
57	Protein ORLA 18 (Protein A)	ORLA Proteins ORLA 18
58	Protein ORLA 85 (Protein G)	ORLA Proteins ORLA 85

Table S3. Primary and secondary antibodies used for immunofluorescence, related to immune-fluorescent staining in Experimental Procedures .

**Primary Antibody [Catalogue Number]**	**Species**	**Supplier of Primary Antibody**	**Secondary Antibody [Catalogue number]**	**Supplier of Secondary Antibody**
Albumin (ALB) [A80-129A]	Goat	Bethyl	Alexa 647 Donkey Anti-Goat [A21447]	Thermo Scientific
A1AT [HM2358]	Mouse	Hycult	Alexa 488 donkey anti mouse [R37114]	Thermo Scientific
Alpha-fetoprotein (AFP) [AB3980]	Mouse	Abcam	Alexa 568 Donkey Anti-Mouse [A10037]	Thermo Scientific
CYP2A6 [Nil]	Hen	Collaborator (University of Eastern Finland)	Alexa 488 Donkey Anti-Hen [703-545-155]	Jackson Immuno-Research Laboratories
HNF4α [AB92378]	Rabbit	Abcam	Alexa 568 Donkey Anti-Rabbit [A10042]	Thermo Scientific

Table S4. Human Taqman probe and primers for RT-PCR analysis were from Thermo Fisher Scientific, related to Real-Time PCR analysis in Experimental Procedures

Target	Cat	Dye
ACTB (Housekeeper)	Hs01060665_g1	VIC
ALBUMIN	Hs00609411_m1	FAM
HNF4A	Hs00230853_m1	FAM
ASGR1	Hs01005019_m1	FAM
CEBP/A	Hs00269972_s1	FAM
CYP1A1	Hs01054797_g1	FAM
CYP2C9	Hs02383631_s1	FAM

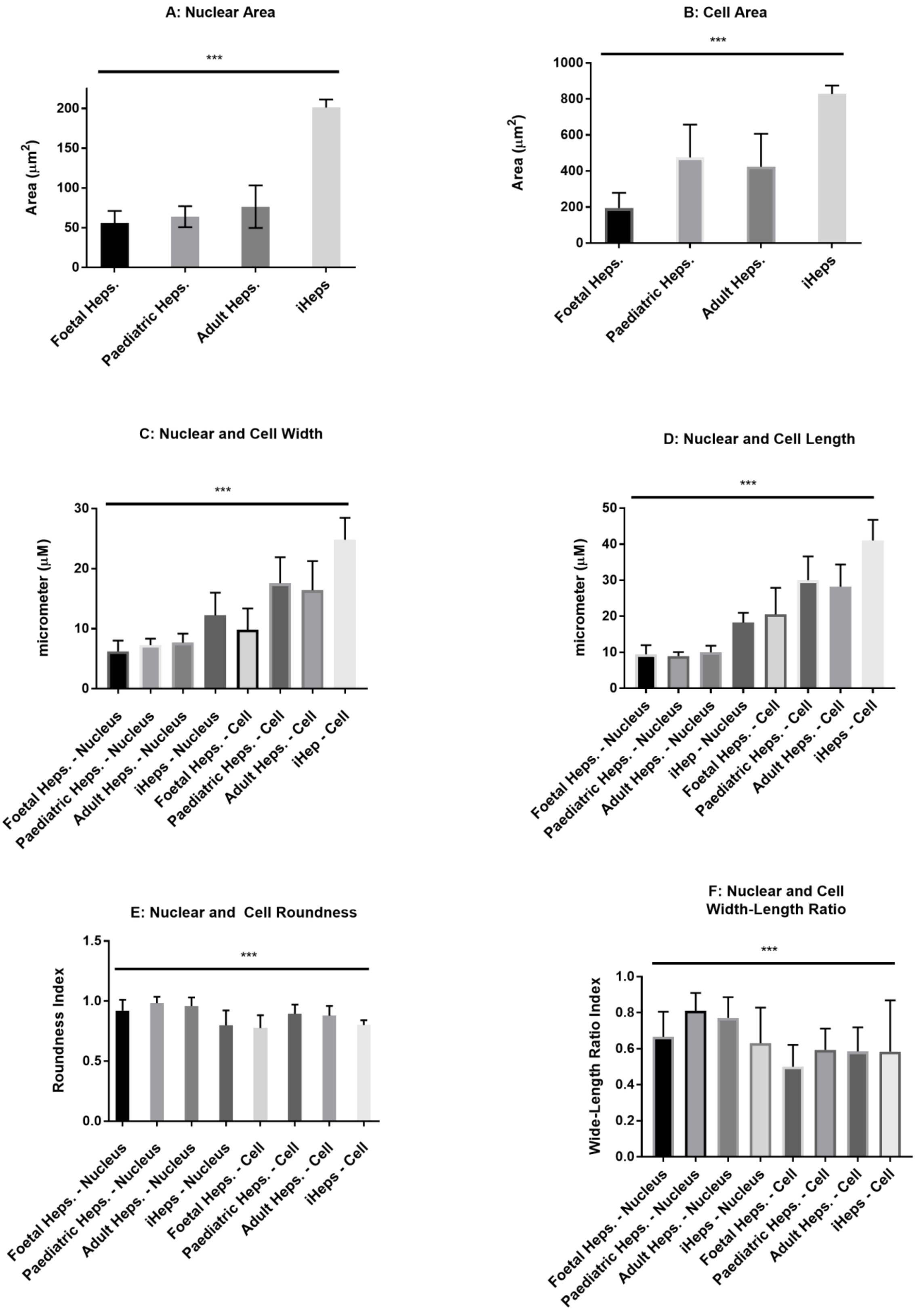

Figure S1. Measured morphological parameters of human hepatocytes by A: nuclear area, B: cell area, C: nuclear and cell width, D: nuclear and cell length, E: nuclear and cell roundness and F: nuclear and cell width-length ratio, related to text in Results section.

(A)

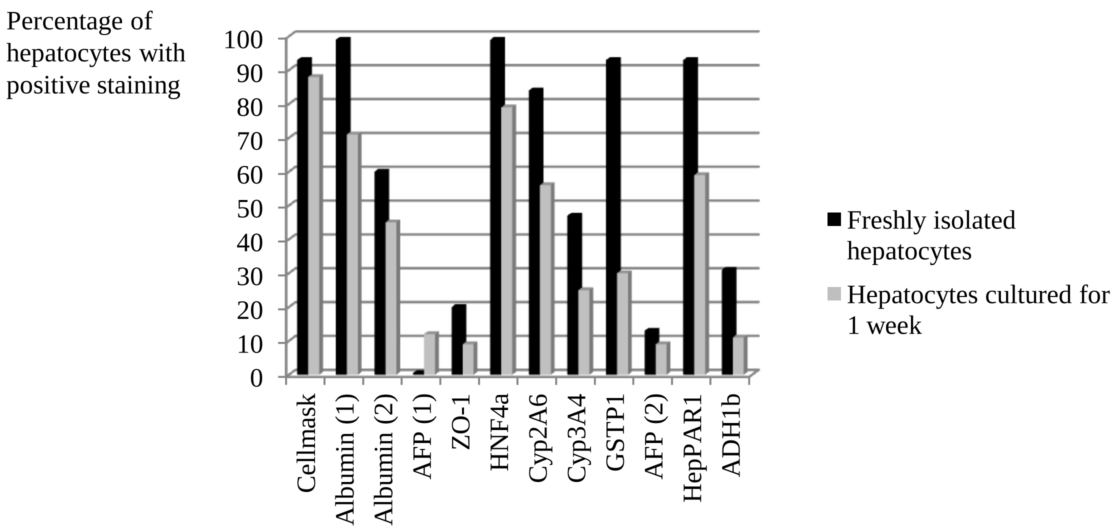

(B)

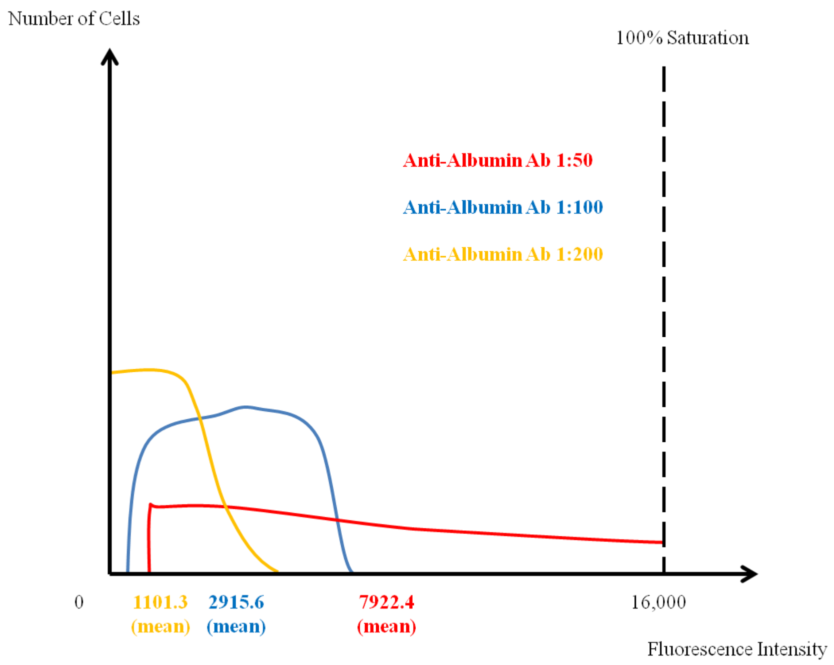

Excitation was fixed at 100% and exposure time was 200ms

Increasing excitation and exposure times stretched the graphs towards the right and caused more cells to approach 100% saturation.

Decreasing excitation and exposure times resulted in a left shift of the graphs towards under-capture of hepatocyte populations.

Figure S2. (A) Performance of various immuno-flourescent antibodies evaluated to screen for hepatocyte likeness (B) High concentrations of Albumin antibody (1:50) resulted in a proportion of cells with 100% saturation. Low concentrations of Albumin antibody (1:200) resulted in under-capture of the cell population with weak staining in a significant proportion of hepatocytes. The optimum concentration of albumin antibody was found to be 1:100. All experiments were performed on 3 separate populations of adult hepatocytes. Figure S2 is related to the text in the Results section.

(A)

Identification of all cell by nuclear staining

(1) Find Nuclei by DAPI
(2) Optimise nuclear segmentation
(3) Measure nuclear properties:
 (a) Nuclear Width
 (b) Nuclear Length
 (c) Nuclear Wide-Length Ratio
 (d) Nuclear Area
 (e) Nuclear Roundness

⇩

Identification of all cell types by cellular staining

(1) Find cytoplasm by albumin
(2) Optimise cell segmentation to include polyploid cells (yellow arrows)
(3) Measure cell properties:
 (a) Cell Width
 (b) Cell Length
 (c) Cell Wide-Length Ratio
 (d) Cell Area
 (e) Cell Roundness
 (f) Mean fluorescence intensity

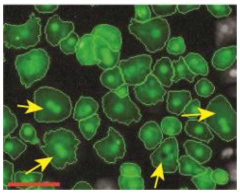

⇩

Automated Selection of "Positive" Hepatocytes by:

(1) Nuclear properties within user-defined range, AND
(2) Cellular properties within user-defined range, AND
(3) Cytoplasmic Mean Fluorescence Intensity within user-dfined range

⇩

Calculation of Hepatocyte Likeness Index (HLI):

(1) Calculate total number of "Positive" Hepatocytes
(2) Calculate total number of cells indentified by Nuclei
(3) Calculate HLI by dividing (1) by (2)

⇩

Other Useful Outputs and Applications::

1) Hepatocyte specific analyses in co-cultures
2) Assessing response of hepatocytes to drugs/compounds
3) Gauging maturity of hepatic progenitor cells
4) Assessing functionality of cryo-preserved hepatocytes

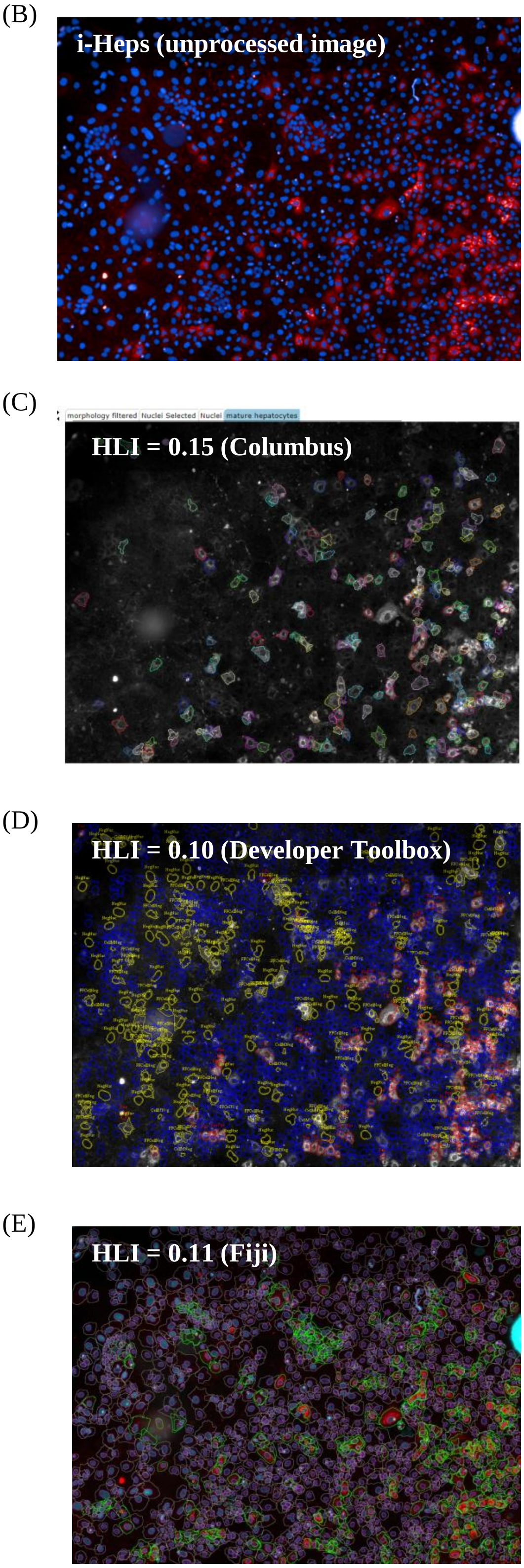

Figure S3. (A) Steps in developing our (Columbus) pipeline using "building blocks" for the derivation of the Hepatocyte Likeness Index; HLI. (B) Unprocessed, reference image of i-Heps that the following pipelines were tested against: (C) Columbus pipeline identifies and highlights mature hepatocytes in grey. (D) Developer Toolbox pipeline identifies mature hepatocytes and highlights these cells in red. (E) ImageJ pipeline identifies mature hepatocytes and highlights these cells in green. (D) and (E) are prototype pipelines created to demonstrate the reproducibility of our Columbus algorithm. (B) to (E) is a selected image from 3 experimental replicates using i-Heps derived from n=3 human iPS cell lines. Pipelines available upon request. Figure S3 is related to text in Results section.

Figure S4. Forward and reverse primers designed for RT-qPCR using PrimerQuest tool (Integrated DNA Technologies), related to Real Time PCR analysis in Experimental Procedures section . The sequences were analyzed using BLAST for specificity for the target sequence at used at 250nM concentration. ALB: albumin, AFP: α-fetoprotein, ACTB: β-actin, FOXA1: forkhead box protein A1, CYP3A4: cytochrome P3A4, HNF4A: hepatocyte nuclear factor 4 alpha,

Target	Forward (5'-3')	Reverse (5'-3')
**ALB**	CGTCGAGATGCACACAAGA	GATACTGAGCAAAGGCAATCAAC
**AFP**	TGAATCCAGAACACTGCATAGAA	TATGGTAGCCAGGTCAGCTA
**ACTB**	CTTCCTTCCTGGGCATGG	GTACAGGTCTTTGCGGATGT
**FOXA1**	AGGGCTGGATGGTTGTATTG	GAGTAGGCCTCCTGCGT
**CYP3A4**	CCTGGTGCTCCTCTATCTATATG	AGCCCTTATGGTAGGACAAA
**HNF4A**	ACATGTACTCCTGCAGATTTAG	CCCGGAAGCATTTCTTGA
