# Supplementary material for: Imaging-Based Screen Identifies Laminin 411 as a Physiologically Relevant Niche Factor with Importance for i-Hep Applications
Source: Stem Cell Reports. 2018 Mar 1;10(3):693–702. doi: 10.1016/j.stemcr.2018.01.025 (PMC5919292; doi:10.1016/j.stemcr.2018.01.025)
Supplement: Document S2. Article plus Supplemental Information [file mmc2.pdf]

Stem Cell Reports
Report
OPEN ACCESS

Imaging-Based Screen Identifies Laminin 411 as a Physiologically Relevant Niche Factor with Importance for i-Hep Applications

John Ong,[1,6] Maria Paola Serra,[1,6] Joe Segal,[1] Ana-Maria Cujba,[1] Soon Seng Ng,[1] Richard Butler,[2] Val Millar,[3] Stephanie Hatch,[3] Salman Zimri,[1] Hiroyuki Koike,[4] Karen Chan,[4] Andrew Bonham,[4] Michelle Walk,[5] Ty Voss,[5] Nigel Heaton,[1] Ragai Mitry,[1] Anil Dhawan,[1] Daniel Ebner,[3] Davide Danovi,[1] Hiromitsu Nakauchi,[2,7] and S. Tamir Rashid[1,2,7,*]
[1]Centre for Stem Cells and Regenerative Medicine & Institute for Liver Studies, King's College London, London SE1 9RT, UK
[2]The Gurdon Institute Imaging Facility, Cambridge University, Cambridge CB2 1QN, UK
[3]Target Discovery Institute, Oxford University, Oxford OX3 7FZ, UK
[4]Institute for Stem Cell Biology and Regenerative Medicine, Stanford University School of Medicine, Stanford, CA 94305, USA
[5]Perkin Elmer, Houston, TX 77055, USA
[6]Co-first author
[7]Co-Senior author
*Correspondence: tamir.rashid@kcl.ac.uk
https://doi.org/10.1016/j.stemcr.2018.01.025

SUMMARY

Use of hepatocytes derived from induced pluripotent stem cells (i-Heps) is limited by their functional differences in comparison with primary cells. Extracellular niche factors likely play a critical role in bridging this gap. Using image-based characterization (high content analysis; HCA) of freshly isolated hepatocytes from 17 human donors, we devised and validated an algorithm (Hepatocyte Likeness Index; HLI) for comparing the hepatic properties of cells against a physiological gold standard. The HLI was then applied in a targeted screen of extracellular niche factors to identify substrates driving i-Heps closer to the standard. Laminin 411, the top hit, was validated in two additional induced pluripotent stem cell (iPSC) lines, primary tissue, and an *in vitro* model of α1-antitrypsin deficiency. Cumulatively, these data provide a reference method to control and screen for i-Hep differentiation, identify Laminin 411 as a key niche protein, and underscore the importance of combining substrates, soluble factors, and HCA when developing iPSC applications.

INTRODUCTION

Stem cell-derived hepatocytes offer an exciting and novel resource for use in human disease modeling and cell therapy. Both embryonic (induced pluripotent stem cell [PSC]/embryonic stem cell [ESC]) and adult stem cell-derived products remain unsuitable for target downstream applications due to comparatively poor functionality when tested against primary adult counterparts (reviewed in Rashid et al., 2015). Extracellular signals delivered via a complex 3-dimensional protein-rich environment termed the "niche" tightly regulate hepatocyte function (Orford and Scadden, 2008; Morrison and Spradling, 2008). Recent attempts at enhancing the function of hepatocytes derived from iPSCs (i-Heps) by targeting the niche (Shan et al., 2013; Wang et al., 2017), though promising, are still unable to bridge the gap in functionality. One of the major reasons for this failure is the lack of a systematic approach to empirically define a signature of maturity that must be crossed in order for i-Heps to become suitably useful.

The aim of this study was therefore to (1) define a profile of healthy, freshly isolated primary hepatocytes (Hepatocyte Likeness Index; HLI) that cells of interest can be compared against for high-throughput screening (HTS), (2) apply this platform to screen hepatocyte niche factors for their ability to drive i-Heps closer to that target, and (3) validate our findings in a pharma-like screening environment.

RESULTS

High Albumin Expression in Combination with Select Morphological Characteristics Defines a Functionally Relevant Hepatocyte Signature and Enables Calculation of the Hepatocyte Likeness Index

To define the profile of a target healthy hepatocyte, we first measured hepatocyte morphology and protein expression in hepatocytes freshly isolated from normal fetal, neonatal, and adult livers (n = 17, Table S1) and used this information to construct the HLI for comparing other cell types of interest (Figure 1).

Measurement of morphological properties revealed statistically significant differences in all parameters between fetal, pediatric, and adult hepatocytes (Figure S1). True multivariate analysis (Random Forest) identified cell width, cell area, and cell symmetry as the three most important characteristics that differentiated human hepatocytes from non-hepatocyte cell types. Compared

This is an open access article under the CC BY license (http://creativecommons.org/licenses/by/4.0/).

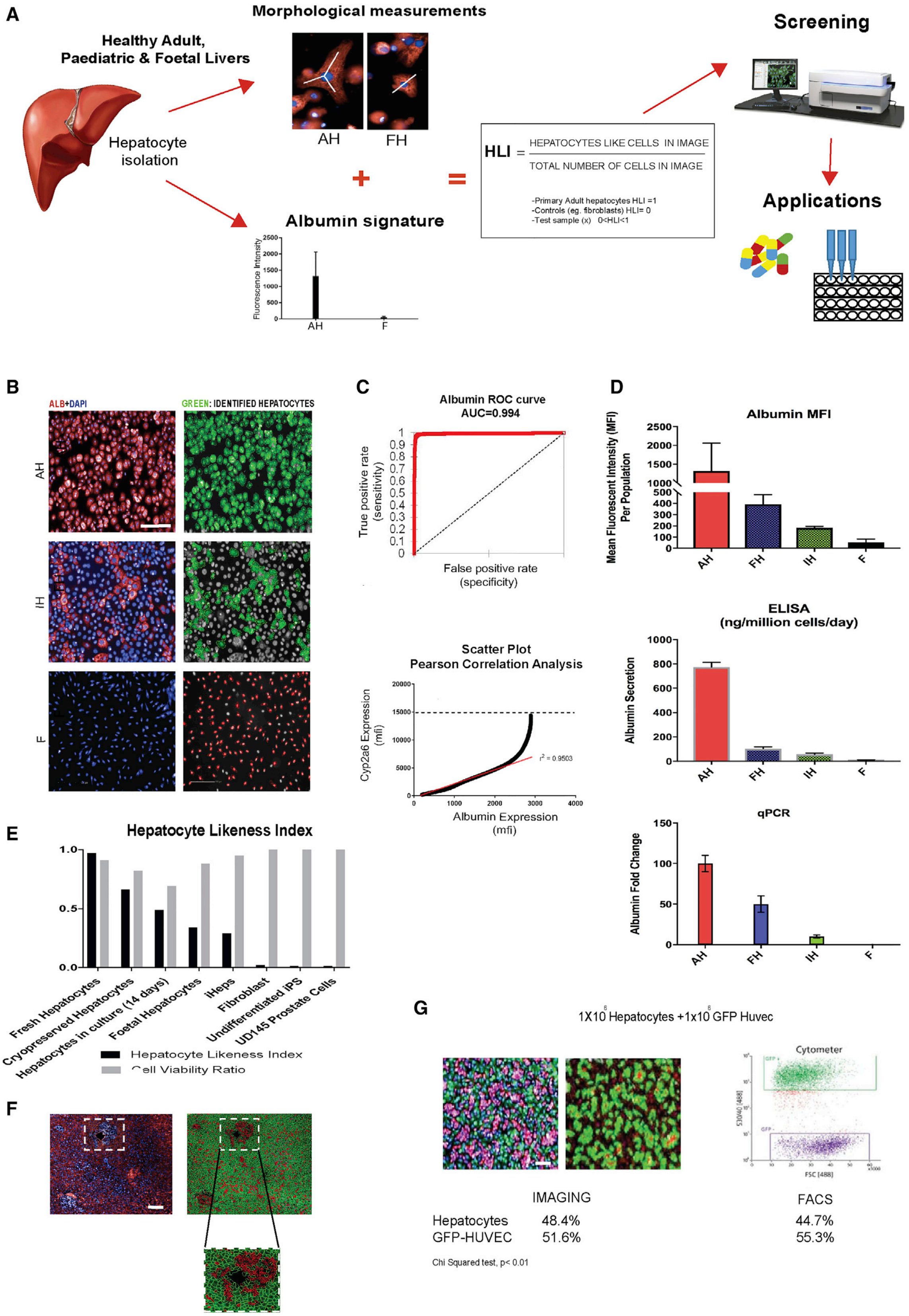

(legend on next page)

with adult hepatocytes, fetal hepatocytes were smaller and less symmetrical. Nuclear area and width also distinguished the physiological age of primary hepatocytes with nuclear polyploidy, as expected, being observed in approximately 30% of hepatocytes from adult liver samples but in less than 5% of cells from fetal liver. i-Heps, on the other hand, resembled primary adult hepatocytes in symmetry but had larger nuclei. Significant variation in their nuclear and cell sizes was also observed, with larger i-Hep morphology correlating poorly with albumin expression. Though promising, the discriminatory ability offered by the above morphological analyses was therefore not enough to proceed to HTS. To improve the accuracy of our index, we therefore evaluated the added value of simultaneously measuring protein expression. Quantification of protein expression by fluorescence intensity was previously widely reported for a broad spectrum of biological applications similar to ours (Young et al., 2008; Massey, 2015; Boehnke et al., 2016; Rodriguez-Muela et al., 2017), making the approach suitable for this study. We therefore tested in different concentrations, exposure times, and excitation levels a panel of immunofluorescent antibodies whose target proteins had previously been reported to correlate with hepatocyte function (Rowe et al., 2013; Baxter et al., 2015) (Figure S2). Analysis of protein expression by mean fluorescence intensity (MFI) in this manner showed discriminatory signatures between i-Heps, fetal hepatocytes, and adult hepatocytes. Albumin expression was found to be the most accurate biomarker for hepatocyte identification in this regard (Figure 1B), correlating best with cellular metabolic function (Figure 1C). The validity of albumin MFI as a surrogate marker for cellular albumin protein level was then confirmed using orthogonal assays such as qPCR and ELISA (Figure 1D). Reassuringly, these experiments demonstrated that increased albumin MFI levels corresponded with relative increases in hepatic function across multiple independent experiments and biological samples.

Incorporating the above data (morphology + albumin MFI), an analysis sequence that enabled supervised machine learning was constructed for the automated identification of bona fide hepatocytes—firstly by nuclear morphology, then cell morphology, and finally albumin expression (Figure S3). Cells satisfying normal parameters (of freshly isolated hepatocytes) were classified as "hepatocyte positive" and others "hepatocyte negative." The HLI was then defined as the proportion of positively identified hepatocytes over the total number of cells analyzed. Therefore, in a population of healthy primary adult hepatocytes HLI approximates 1 and in a negative biological control (e.g. fibroblasts), HLI approximates 0. We next validated the physiological relevance of this index by measuring HLI in different cell populations (Figure 1E) to show how the algorithm could be used to not only differentiate hepatocytes from non-hepatic cells but also to discriminate freshly isolated adult hepatocytes from cryopreserved adult, cultured adult, and freshly isolated fetal hepatocytes in a manner independent of cell viability (Figure 1E). The HLI was also capable of identifying bona fide hepatocytes within heterogeneous cell populations such as is found in human liver tissue (Figure 1F) or in *in vitro* co-culture systems (Figure 1G) with a higher accuracy than flow cytometry. Finally, we demonstrated that the HLI could readily be transferred to other image analysis platforms (Figure S3) before proceeding to screen for the effects of hepatocyte niche factors on i-Hep HLI (Figure 2).

Figure 1. Generation of the Hepatocyte Likeness Index

(A) Schematic representation of work-flow that established morphological parameters and protein signatures for the Hepatocyte Likeness Index (HLI) and its validation across different cell types. AH, adult hepatocytes (n = 17 donors); F, fibroblasts (n = 3 donors).

(B) Image-based algorithm enables the automated identification of hepatocytes by morphology and albumin. (Left column) DAPI and albumin staining in adult hepatocytes (AH), i-Heps (IH), and fibroblasts (F). (Right column) Cells highlighted green represent identified hepatocytes; gray or red highlighted cells are recognized as non-hepatocytes. Scale bar, 200 μm.

(C) (Top) Receiver-operating characteristic curve demonstrating high diagnostic ability of albumin as a surrogate marker of hepatocyte likeness and maturity. (Bottom) Pearson correlation analysis of showing that high albumin expression is closely related with metabolic (cytochrome 2A6) function (Pearson coefficient $r^2 = 0.9503$, 95% confidence interval, $p < 0.01$).

(D) Mean fluorescence intensity (MFI) of albumin is validated by ELISA and qPCR in adult hepatocytes (AH), fetal hepatocytes (FH), i-Heps (IH), and fibroblasts (F). n = 9 donors (AH), n = 4 donors (FH), n = 3 donors (IH), and n = 3 donors (F). Error bars show mean ± SD.

(E) HLI discriminates freshly isolated adult hepatocytes from cryopreserved adult, cultured adult, freshly isolated fetal hepatocytes, and i-Heps in a manner independent of cell viability (n = 3 different biological samples in all groups, all independent experiments).

(F) HLI discriminates hepatocytes from other cells *in vivo*. (Left panel) Human liver section (10×) stained for albumin (red) demonstrating albumin-positive and albumin-negative (blue) cells. (Right panel) Application of HLI algorithm enables automated detection of hepatocytes (green) from non-hepatocytes (red) by machine learning. Enlarged square: accurate distribution of hepatocytes and non-hepatocyte cells around periportal area. Scale bar, 100 μm.

(G) HLI discriminates hepatocytes from other cells *in vitro*. Co-culture of GFP-labeled HUVECs (green) and hepatocytes (red) (left panel) were discriminated by HLI (right panel) better than by flow cytometry (right). Scale bar, 100 μm.

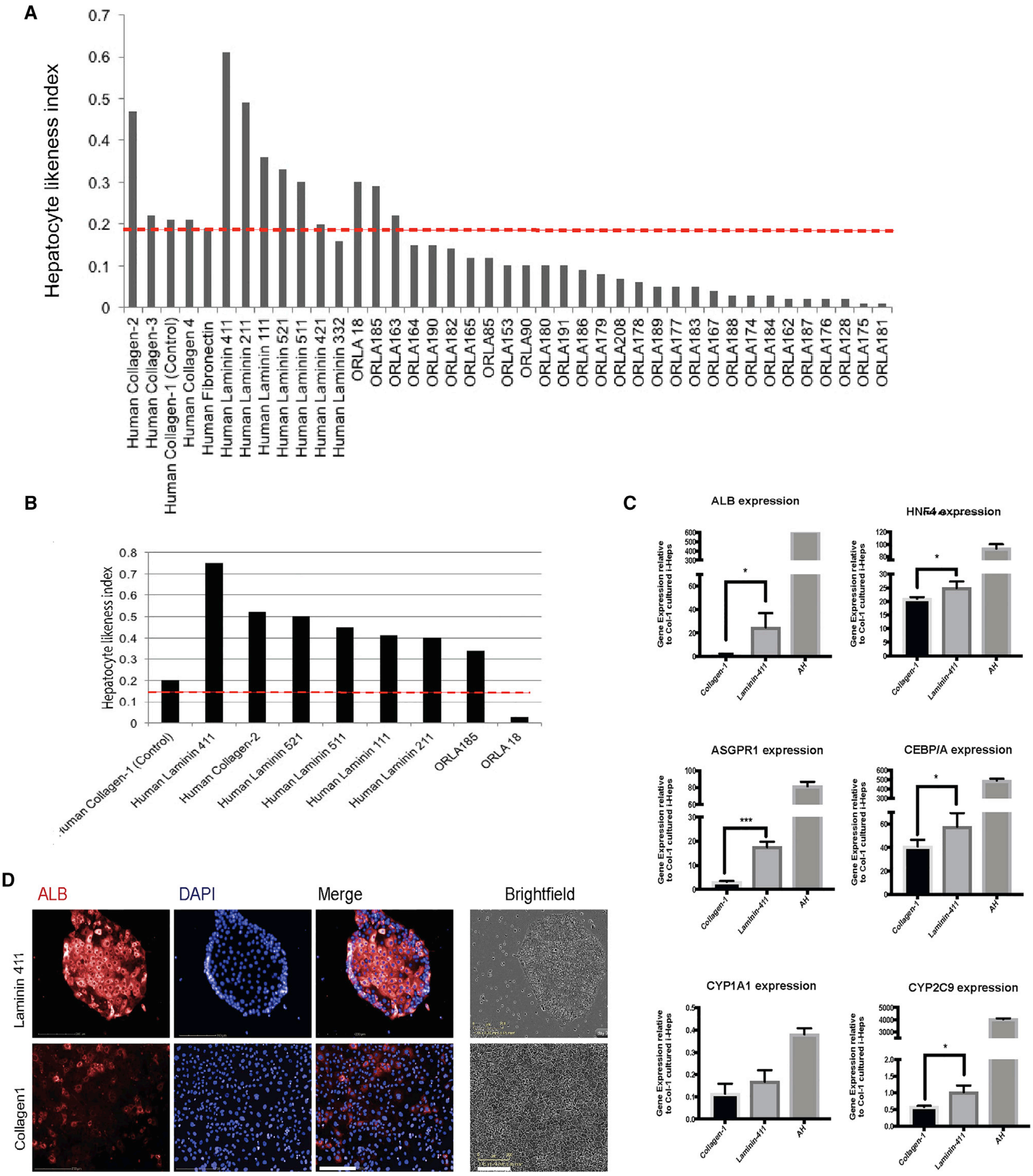

Figure 2. Screening of Niche Factors Using HLI Algorithm Demonstrates Effect of Laminin 411 in i-Heps

(A) The HLI algorithm (y axis) was used to screen for the effects of 58 different hepatocyte niche factors (x axis) on i-Heps 7 days after plating. Control line (red) is the control threshold based on culture with collagen-1.

(B) Revalidation of the eight hits from the first round of screening.

(legend continued on next page)

A Screen of ECM Proteins and Soluble Niche Factors Demonstrates that Laminin 411 Advances i-Heps toward Functional Significance

Using the Human Matrisome Project database (http://matrisomeproject.mit.edu) we identified 105 proteins likely to be important in hepatocyte maturation. Of these, a total of 58 proteins and niche factors were put forward into the screen based on biological interest and commercial availability (Table S2). From the initial screen, eight proteins (Figure 2A) were found to have a positive effect (HLI > 0.2) and taken forward for validation. Seven of the eight proteins were found to be efficacious in the second round (Figure 2B). The hit with the greatest effect on HLI, Laminin 411, was then tested on i-Heps from three different biological samples. In these conditions, cells displayed higher expression levels of genes known to be associated with adult hepatocyte function such as *CYP2C9*, *ASGPR1*, *HNF4A*, *CEBP/A*, and *ALB* (Figure 2C). Finally, immunofluorescence staining for albumin confirmed that i-Heps cultured in Laminin 411 for 2 weeks have higher protein expression with more cells meeting morphological parameters of a normal hepatocyte (Figure 2D).

Laminin 411 Is a Component of the Hepatic Niche in Human Fetal Liver

Next, we investigated the importance of Laminin 411 during human liver development. We obtained freshly isolated human fetal hepatocytes from 16- to 20-pcw (post-coital weeks) donor tissue (n = 3 donors) and observed similar effects of culturing these cells on Laminin 411 as with i-Heps. Compared with collagen-1, Laminin 411 improved cell survival and morphology (Figure 3A) while retaining a higher population of cells mirroring the adult hepatocyte phenotype (Figure 3B). Gene expression analysis confirmed a statistically significant increase in the expression of *ALB*, *HNF4A*, *CYPA1*, and *CYP2C9* (Figure 3C). We then hypothesized that if Laminin 411 is relevant to human physiology of hepatocytes, it would also be expressed in liver. For this purpose, we analyzed gene expression databases for genes expressing ECM proteins in adult versus fetal versus iPSC-endoderm tissue. This analysis demonstrated upregulation of *LAMA4* and *LAMB1* (the constituent components of LAM-411) in human fetal liver (Figure 3D). We confirmed this computational presumption using RNA *in situ* hybridization, and found high expression of *LAMA4* near vascularized regions of maturing human fetal liver and only very weak expression in adult liver (Figure 3E).

Laminin 411 Prolonged Survival of Hepatic Progenitor Cells and Is Better Suited for i-Hep-Based Drug-Screening Applications

Lastly we sought to examine whether our findings had identified a substrate more suitable for use with an iPSC α1-antitrypsin deficiency (A1AT-Z) disease model drug-screening platform (Yusa et al., 2011). Cells were seeded into 384-well plates coated with collagen-1 or Laminin 411 and evaluated for survival, disease signature, inter-well variability, and response to the histone deacetylase (HDAC) inhibitor suberoylanilide hydroxamic acid (SAHA) (Bouchecareilh et al., 2012). After 28 days in culture (Figure 4A), Laminin 411 demonstrated improved cell survival and had a stronger disease signature of A1AT (Figure 4B) compared with collagen-1 when stained for albumin and A1AT protein. Improvement in cell survival was also observed in primary fetal hepatocytes; however, similarly to current literature this effect was not seen in primary adult hepatocytes (Watanabe et al., 2016).

DISCUSSION

Here, we report the development of an imaging-based algorithm capable of stratifying human hepatocyte maturity in a high-throughput manner. With this capability, candidate extracellular niche factors were screened for their capacity to drive i-Heps closer to hepatocytes freshly isolated from human liver. The efficacy of the most significant hit, Laminin 411, was confirmed in three different iPSC lines and three different human fetal hepatocyte samples. Translational relevance was then demonstrated by superior performance of the substrate in a high-throughput drug-screening platform for α1-antitrypsin deficiency.

Imaging-based assays represent an excellent modality for studying the effects of extracellular niche components in a high-throughput manner. Bhatia and colleagues recently reported the development of an exciting high-throughput, imaging-based genetic screening platform to identify the most critical stromal cell gene products involved in stabilization of primary human hepatocyte functions (Shan et al., 2016). Our work extends the significance of such studies by using freshly isolated hepatocytes, the physiological gold

(C) Relative gene expression of i-Heps cultured on Laminin 411 (middle bars) compared with collagen-1 (left bars) with expression levels in freshly isolated adult hepatocytes (AH) as control (right bars). n = 3 (different i-Hep cell lines and independent experiments); error bars show mean ± SD; *p < 0.05, ***p < 0.01.

(D) Immunofluorescence staining for albumin (red, left), DAPI (blue, left middle) and merge (middle right), of i-Heps cultured on Laminin 411 (top) versus collagen-1 (bottom); cell morphology is shown on far right (10× magnification; scale bar, 100 μm). Images shown represent n = 3 different biological replicates and independent experiments. Scale bar, 200 μm.

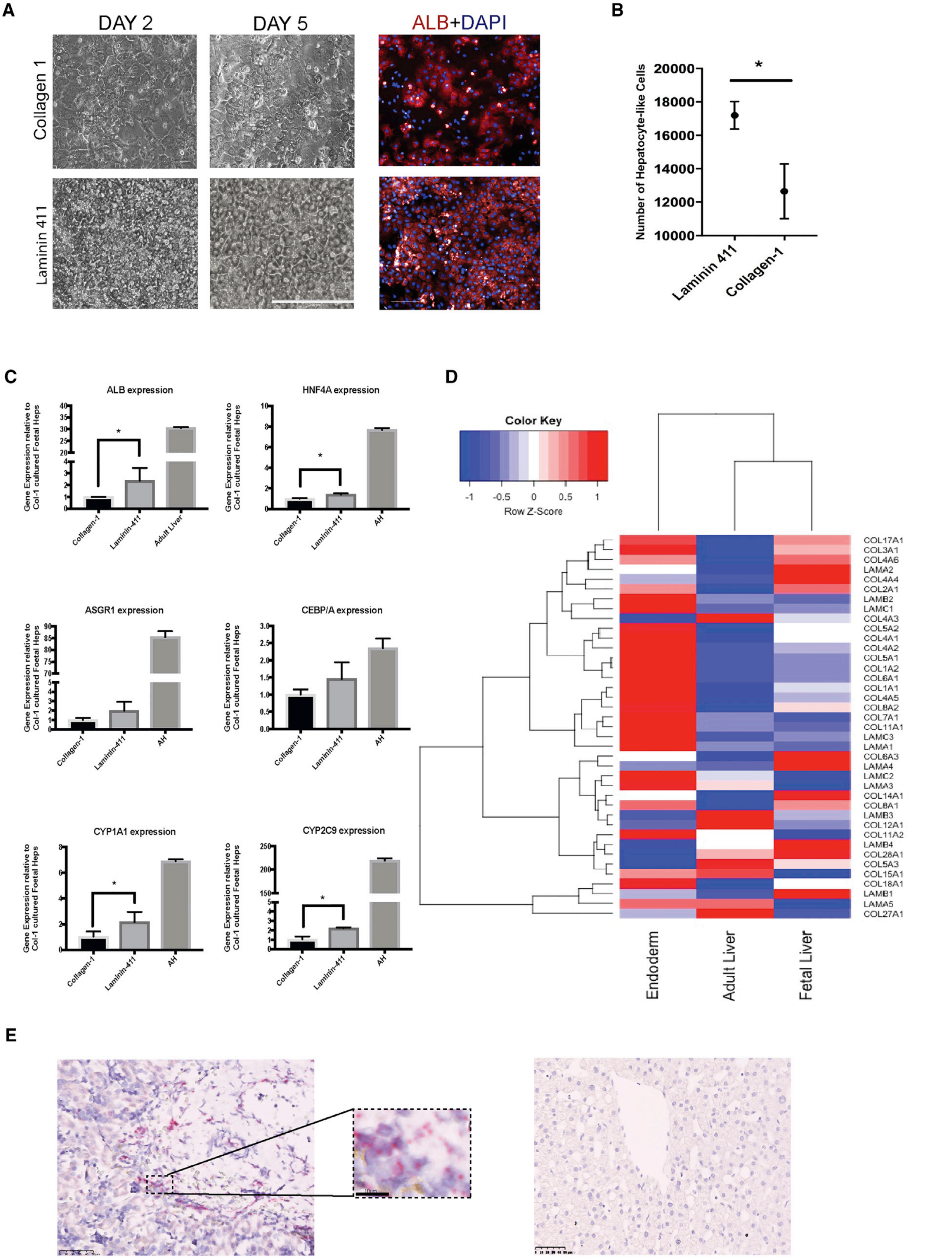

(legend on next page)

standard in the field (instead of frozen cells) to construct a "hepatocyte-ness" score (the HLI) for measuring the effects of substrates on stem cell-derived hepatocytes. These tools are, we believe, essential for expediting effective translational outputs in this field, with the generalizability of the platform (reproducibility across multiple image analysis platforms, cell lines, and commercially available substrates) suggesting that rapid adaptation by others could easily be achieved.

Results from the screen performed highlighted the important role played by laminins, a group of heterotrimeric ECM proteins, in the hepatic progenitor response. This association had previously been reported in adult liver (Kallis et al., 2011) and in cholangiocyte differentiation (Takayama et al., 2016) but not in embryonic hepatocyte development. Here we demonstrated Laminin 411 to be a biologically relevant and important factor in advancing i-Hep differentiation and in human hepatic fetal development (Figure 3). Although its specific role remains unclear, its structure comprising α-4 (LAMA4), β-1, and γ-1 chains, facilitates binding to $\alpha_6\beta_1$ and $\alpha_6\beta_4$ integrin receptors, suggesting that its effects may be due to activation of several receptors or pathways yet to be fully elucidated. This heterogeneity opens up the likelihood of downstream function being a consequence of co-engagement with a combination of as yet poorly defined soluble and insoluble factors. Our future efforts will therefore seek to unravel these complex interactions by combining soluble factors with insoluble ECM proteins in more sophisticated HTS screens. In the meantime, as shown by our A1AT drug-screening data (Figure 4), information from even the most basic of screens using the algorithm can rapidly be translated into meaningful advances, which in turn suggests that our approach could be of widespread utility in the field.

EXPERIMENTAL PROCEDURES

Cells

All human tissues were collected with informed consent following ethical and institutional guidelines. Freshly isolated hepatocytes were obtained from Triangle Research Labs, the Institute of Liver Studies (King's College Hospital), while fetal livers were obtained from the Human Developmental Biology Resource of University College London. Isolated adult hepatocytes were plated on 96-well collagen-1-coated plates (Greiner) at a density of 10,000 cells per well and maintained in commercially supplied media. Fetal hepatocytes were obtained from 16- to 20-week-old fetuses, dissociated as previously described (Gramignoli et al., 2012) and cultured in collagen-1 or Laminin 411 (Biolamina, 10 μg/mL) while being maintained on DMEM supplemented with insulin (Sigma) and dexamethasone (Sigma) (10^{-7} M).

Human i-Heps were obtained from Definigen as a cryopreserved sample and recovered for 10 days as per manufacturer's instructions (ref. 16; A, patient 2 line 1; B, patient 1 line 1; C, patient 3 line 1) or supplied from the Nakauchi lab (TkDA line as used in Takebe et al., 2013). Cells were plated on flat-bottomed 96-well plates pre-coated with collagen-1 or Laminin 411 (as previously described) at a density of 1.25×10^5 cells per well. Cultures were maintained in Hepatozyme medium (Life Technologies), supplemented with 1% L-glutamine, 1% penicillin/streptomycin, 2% non-essential amino acids (Life Technologies), 2% lipid concentrate (Life Technologies), 0.1% insulin-transferrin-selenium (Sigma), 0.01 μg/mL oncostatin M (Peprotech), and 0.05 μg/mL hepatocyte growth factor (Peprotech).

For the co-culture system, hepatocytes were cultured in a 1:1 ratio with nuclear GFP-labeled human umbilical vein endothelial cells (HUVECs) (Essen Bioscience) using a 1:1 mix of Hepatozyme and EGM (Lonza).

Human fibroblasts from three donors were isolated from skin explants dissociation and cultured in collagen-coated 96-well plates at a density of 500,000 cells/mL.

Cell viability was determined by trypan blue (Invitrogen) exclusion and all cells were fixed 4% (w/v) paraformaldehyde phosphate buffer solution (PFA; Sigma) after 48 hr of plating.

ECM Screening

Proteins were used individually, in pairs, or in combinations of three at five different concentrations by overnight coating of 96-well plates. Concentrations used for coating were dependent on the target protein. I-Heps were plated for 7, 14, and 28 days before being fixed, stained, and imaged as per the protocol defined earlier. The most significant hits from the first round were taken

Figure 3. Laminin 411 Is a Physiologically Relevant Niche Factor in Fetal Liver Development

(A) Morphology (20×) of fetal hepatocytes cultured on collagen-1 (top) versus Laminin 411 (bottom) at 2 (left) and 5 (middle) days post plating (scale bar, 200 μm). Immunofluorescence staining (right) for albumin (red) plus DAPI (blue) at 5 days post plating (scale bar, 100 μm).

(B) Number of albumin-expressing fetal hepatocytes identified by HLI algorithm (y axis), cultured on Laminin 411 versus collagen (x axis) at day 5. n = 3 different biological samples and independent experiments; data presented as mean ± SD; *$p < 0.05$.

(C) Relative gene expression (y axis) of fetal hepatocytes cultured for 7 days on Laminin 411 (middle bars) versus collagen 1 (left bars) compared with adult liver (right bars). n = 3 (different biological samples and independent experiments); data presented as mean ± SD; *$p < 0.05$.

(D) ECM gene expression heatmap comparing iPSC-derived endoderm (left) with adult (middle) and fetal (right) liver.

(E) RNA *in situ* hybridization for *LAMA4* on a 16-pcw liver slide (left) (40× magnification; scale bar, 50 μm). Detail of the red dots expanded in the square compared with adult liver (right).

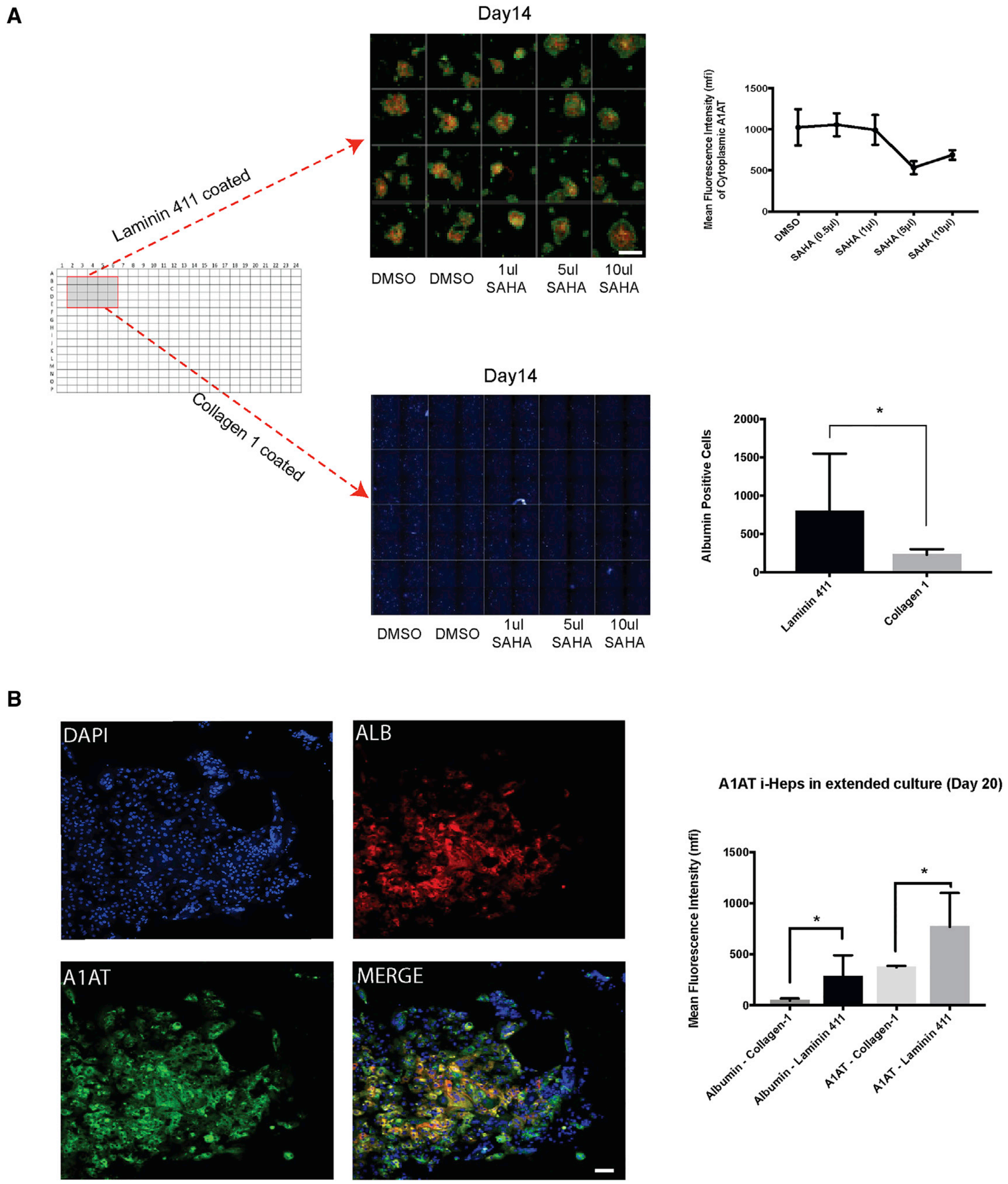

Figure 4. Laminin 411 Is Better Suited for Drug Screening in an i-Hep Disease Model of α1-Antitrypsin Deficiency

(A) I-Heps plated on 384-well plates coated with Laminin 411 or collagen-1 (left) were cultured for 14 days then imaged using the HLI (scale bar, 1 mm) i-Heps cultured on Laminin 411 responded to SAHA treatment (top right) and have more function than albumin-producing cells (bottom right). Data presented as mean ± SD; n = 3 biological replicates from independent experiments; $*p < 0.05$.

(B) Enlarged images from (A) showing immunofluorescence staining of i-Heps, DAPI (blue top left), albumin (red top right), A1AT (green bottom left), and MERGE (yellow bottom right; scale bar, 50 μm). MFI for images were quantified (right graph) and showed higher MFI of both A1AT and albumin in Laminin 411 (middle bars) compared with collagen-1 (outer bars). Data presented as mean ± SD; n = 3 biological replicates from independent experiments; $*p < 0.05$.

forward into a secondary screen using two additional iPSC cell lines generated using two separate protocols (Rashid et al., 2010; Mallanna and Duncan, 2013).

Image Analysis

Staining with CellMask blue was used for cell segmentation, images captured with Operetta (PerkinElmer), and analysis

performed by PhenoLOGIC software (PerkinElmer) to identify parameters that co-relate most closely with physiological hepatocyte likeness. Albumin staining in primary hepatocytes was found to be equal to CellMask blue for cell segmentation. Areas under receiver-operating characteristic curves were plotted using XLSTAT and Prism (GraphPad) to determine the optimum cutoff fluorescent intensities.

For the 384-well plates, images were taken with a 10× objective on the GE IN Cell Analyzer 6000.

Immunofluorescent Staining

After fixation, cells were blocked and permeabilized in 1% (w/v) BSA (Sigma-Aldrich), 3% donkey serum (Life Technologies), and 0.1% Triton X-100. Primary antibodies (Table S3) were applied for 1 hr. After washes, cells were incubated with Alexa 647, Alexa 568, Jackson 488-conjugated secondary antibodies (Life Technologies). Slides were counterstained with DAPI (NucBlue) (Life Technologies).

Paraffin-embedded sections (5 μm thickness) were dewaxed, rehydrated, and subjected to an antigen retrieval procedure with sodium citrate (Sigma) (pH 6.0) before following the staining procedure as described above.

Real-Time PCR Analysis

Total RNA was harvested using TRI reagent (Sigma), treated with DNase (Promega), and phenol/chloroform purified. For each sample 0.5 μg of total RNA was reverse transcribed using a SuperScript VILO cDNA Synthesis kit (Thermo Fisher Scientific). A typical RT-PCR reaction contained 10 ng of sample cDNA, 0.0075 μL of individual forward and reverse primer each at 100 μM stock, 5 μL of Taqman Universal Master mix (Applied Biosystems), 1 μL of Taqman target probe (Table S4), and made up to 10 μL with nuclease-free water. Real-time PCR reactions were amplified for 40 cycles on a CFX384 Touch Real-Time PCR Detection System (Bio-Rad) in triplicate and normalized to ACTB (Figure S4) in the same run. Real-time RT-PCR data are presented as the mean of three independent biological experiments, and error bars indicate SEM.

Heatmap Generation

Heatmaps were generated from bulk RNA-sequencing data collected from three human fetal livers (two 14-pcw samples and one 16 pcw), three human adult livers (female 18 years, male 13 years, and female 36 years), and three i-Hep samples harvested at day 6 (endodermal stage) from the BOB cell line. Starting with 1 μg input total RNA, rRNA was removed using a Ribo-Zero Gold rRNA Removal kit (Illumina). Sequencing libraries were prepared using NEBNext Ultra Directional RNA Library Prep Kit for Illumina (NEB) using 100 ng of rRNA-depleted sample and sequenced on a HiSeq 2500 system in rapid run mode (Illumina) following a standard protocol. All libraries generated between 15 and 25 million reads. Reads were mapped to GRCh38 reference genome using Bowtie2 (Langmead and Salzberg, 2012). Raw counts and normalized gene expression were generated using HT-Seq (Anders et al., 2015) and DESEq2 (Love et al., 2014) packages, respectively. The heatmap was generated using R (http://www.R-project.org/) (R Development Core Team, 2008). Heatmaps represent average DESEq2 normalized gene expression values of three independent biological samples.

Human Albumin ELISA

Albumin production of all different cell types was assessed using the Human Albumin Quantification Set (Bethyl Laboratories) following the manufacturer's instructions. Results shown are the mean and SD of triplicate biological replicates. Absorbance was measured using a GloMax-Multi Detection System (Promega) at 450 nm.

In Situ Hybridization

In situ hybridization for *LAMA4* (Hs-LAMA4, cat. No. 459161) expression was performed on 5-μm sections of 15- to 17-pcw fetal and adult livers using the RNAscope 2.5 High Definition (Red, cat. no. 322350, Advanced Cell Diagnostics, Hayward, CA) according to the manufacturer's instructions. Slides were counterstained with Gill's hematoxylin and analyzed under the Hamamatsu scanner.

Data Analysis

Data were analyzed with GraphPad Prism for Pearson Correlation Analyses and t tests. t Tests were plotted as mean ± SD or mean ± SEM where specified. Phenologic and Spotfire (PerkinElmer) were also used for multivariate analysis (Random Forrest), PCA plot, and SOM algorithm.

SUPPLEMENTAL INFORMATION

Supplemental Information includes four figures and four tables and can be found with this article online at https://doi.org/10.1016/j.stemcr.2018.01.025.

AUTHOR CONTRIBUTIONS

S.T.R., H.N., and J.O. designed the experiments, which were carried out by J.S., A.-M.C., S.S.N., R.B., V.M., S.H., S.Z., H.K., K.C., A.B., M.W., T.V., N.H., R.M., A.D., D.E., and D.D., led by J.O. and M.P.S. All authors contributed to data analysis and writing of the manuscript, led by J.O., M.P.S., H.N., and S.T.R.

ACKNOWLEDGMENTS

The research was funded/supported by (1) the National Institute for Health Research (NIHR) Clinical Research Facility at Guy's & St Thomas' NHS Foundation Trust and NIHR Biomedical Research Centre based at Guy's and St Thomas' NHS Foundation Trust and King's College, London; and (2) the UKRMP Niche Platform.. The views expressed are those of the author(s) and not necessarily those of the NHS, the NIHR, or the Department of Health. M.P.S. received funding from the European Union's Horizon 2020 Research and Innovation Program under the Marie Sklodowska-Curie grant agreement number 705607 and the EASL postdoctoral fellowship scheme. J.O. received additional funding from the Royal College of Physicians through the award of the Dame Sheila Sherlock Traveling Scholarship. H.N. is supported by CIRM. S.T.R. is supported by the MRC via a Clinician Scientist Fellowship award MGSBACR. We would also like to thank Prof. Fiona Watt, Dr. Aamir Ahmed,

Simon Broad, and Natalia Palasz for reagents and support. S.T.R. is a founder and shareholder in DefiniGEN.

Received: August 18, 2017
Revised: January 21, 2018
Accepted: January 22, 2018
Published: February 22, 2018

REFERENCES

Anders, S., Pyl, P.T., and Huber, W. (2015). HTSeq—a Python framework to work with high-throughput sequencing data. Bioinformatics *31*, 166–169.

Baxter, M., Withey, S., Harrison, S., Segeritz, C.P., Zhang, F., Atkinson-Dell, R., Rowe, C., Gerrard, D.T., Sison-Young, R., Jenkins, R., et al. (2015). Phenotypic and functional analyses show stem cell-derived hepatocyte-like cells better mimic fetal rather than adult hepatocytes. J. Hepatol. *62*, 581–589.

Bouchecareilh, M., Hutt, D.M., Szajner, P., Flotte, T.R., and Balch, W.E. (2012). Histone deacetylase inhibitor (HDACi) suberoylanilide hydroxamic acid (SAHA)-mediated correction of α1-antitrypsin deficiency. J. Biol. Chem. *287*, 38265–38278.

Boehnke, K., Iversen, P.W., Schumacher, D., Lallena, M.J., Haro, R., Amat, J., Haybaeck, J., Liebs, S., Lange, M., Schäfer, R., et al. (2016). Assay establishment and validation of a high-throughput screening platform for three-dimensional patient-derived colon cancer organoid cultures. J. Biomol. Screen. *21*, 931–941.

Gramignoli, R., Green, M.L., Tahan, V., Dorko, K., Skvorak, K.J., Marongiu, F., Zao, W., Venkataramanan, R., Ellis, E.C., Geller, D., et al. (2012). Development and application of purified tissue dissociation enzyme mixtures for human hepatocyte isolation. Cell Transpl. *21*, 1245–1260.

Kallis, Y.N., Robson, A.J., Fallowfield, J.A., Thomas, H.C., Alison, M.R., Wright, N.A., Goldin, R.D., Iredale, J.P., and Forbes, S.J. (2011). Remodelling of extracellular matrix is a requirement for the hepatic progenitor cell response. Gut *60*, 525–533.

Langmead, B., and Salzberg, S. (2012). Fast gapped-read alignment with Bowtie 2. Nat. Methods *9*, 357–359.

Love, M.I., Huber, W., and Anders, S. (2014). Moderated estimation of fold change and dispersion for RNA-seq data with DESeq2. Genome Biol. *15*, 550.

Mallanna, S.K., and Duncan, S.A. (2013). Differentiation of hepatocytes from pluripotent stem cells. Curr. Protoc. Stem Cell Biol. *26*, Unit 1G.4.

Massey, A.J. (2015). Multiparametric cell cycle analysis using the operetta high-content imager and harmony software with PhenoLOGIC. PLoS One *10*, e0134306.

Morrison, S.J., and Spradling, A.C. (2008). Stem cells and niches: mechanisms that promote stem cell maintenance throughout life. Cell *132*, 598–611.

Orford, K.W., and Scadden, D.T. (2008). Deconstructing stem cell self-renewal: genetic insights into cell-cycle regulation. Nat. Rev. Genet. *9*, 115–128.

Rashid, S.T., Corbineau, S., Hannan, N., Marciniak, S.J., Miranda, E., Alexander, G., Huang-Doran, I., Griffin, J., Ahrlund-Richter, L., Skepper, J., et al. (2010). Modeling inherited metabolic disorders of the liver using human induced pluripotent stem cells. J. Clin. Invest. *120*, 3127–3136.

Yusa, K., Rashid, S.T., Strick-Marchand, H., Varela, I., Liu, P.Q., Paschon, D.E., Miranda, E., Ordóñez, A., Hannan, N.R., Rouhani, F.J., et al. (2011). Targeted gene correction of α1-antitrypsin deficiency in induced pluripotent stem cells. Nature *478*, 391–394.

Rashid, T., Takebe, T., and Nakauchi, H. (2015). Novel strategies for liver therapy using stem cells. Gut *64*, 1–4.

Rowe, C., Gerrard, D.T., Jenkins, R., Berry, A., Durkin, K., Sundstrom, L., Goldring, C.E., Park, B.K., Kitteringham, N.R., Hanley, K.P., et al. (2013). Proteome-wide analyses of human hepatocytes during differentiation and dedifferentiation. Hepatology *58*, 799–809.

R Development Core Team. R: A language and environment for statistical computing. R Foundation for Statistical Computing, Vienna, Austria. (2008). ISBN 3-900051-07-0, URL http://www.R-project.org.

Rodriguez-Muela, N., Litterman, N.K., Norabuena, E.M., Mull, J.L., Galazo, M.J., Sun, C., Ng, S.Y., Makhortova, N.R., White, A., Lynes, M.M., et al. (2017). Single-cell analysis of SMN reveals its broader role in neuromuscular disease. Cell Rep *18*, 1484–1498.

Shan, J., Schwartz, R.E., Ross, N.T., Logan, D.J., Thomas, D., Duncan, S.A., North, T.E., Goessling, W., Carpenter, A.E., and Bhatia, S.N. (2013). Identification of small molecules for human hepatocyte expansion and iPS differentiation. Nat. Chem. Biol. *9*, 514–520.

Shan, J., Logan, D.J., Root, D.E., Carpenter, A.E., and Bhatia, S.N. (2016). High-throughput platform for identifying molecular factors involved in phenotypic stabilization of primary human hepatocytes in vitro. J. Biomol. Screen. *21*, 897–911.

Takebe, T., Sekine, K., Enomura, M., Koike, H., Kimura, M., Ogaeri, T., Zhang, R.R., Ueno, Y., Zheng, Y.W., Koike, N., et al. (2013). Vascularized and functional human liver from an iPSC-derived organ bud transplant. Nature *499*, 481–484.

Takayama, K., Mitani, S., Nagamoto, Y., Sakurai, F., Tachibana, M., Taniguchi, Y., Sekiguchi, K., and Mizuguchi, H. (2016). Laminin 411 and 511 promote the cholangiocyte differentiation of human induced pluripotent stem cells. Biochem. Biophys. Res. Commun. *474*, 91–96.

Wang, B., Li, W., Dean, D., Mishra, M.K., and Wekesa, K.S. (2017). Enhanced hepatogenic differentiation of bone marrow derived mesenchymal stem cells on liver ECM hydrogel. J. Biomed. Mater. Res. A https://doi.org/10.1002/jbm.a.36278.

Watanabe, M., Zemack, H., Johansson, H., Hagbard, L., Jorns, C., Li, M., and Ellis, E. (2016). Maintenance of hepatic functions in primary human hepatocytes cultured on xeno-free and chemical defined human recombinant laminins. PLoS One *11*, e0161383.

Young, D.W., Bender, A., Hoyt, J., McWhinnie, E., Chirn, G.W., Tao, C.Y., Tallarico, J.A., Labow, M., Jenkins, J.L., Mitchison, T.J., et al. (2008). Integrating high-content screening and ligand-target prediction to identify mechanism of action. Nat. Chem. Biol. *4*, 59–68.

Stem Cell Reports, Volume *10*

Supplemental Information

Imaging-Based Screen Identifies Laminin 411 as a Physiologically Relevant Niche Factor with Importance for i-Hep Applications

John Ong, Maria Paola Serra, Joe Segal, Ana-Maria Cujba, Soon Seng Ng, Richard Butler, Val Millar, Stephanie Hatch, Salman Zimri, Hiroyuki Koike, Karen Chan, Andrew Bonham, Michelle Walk, Ty Voss, Nigel Heaton, Ragai Mitry, Anil Dhawan, Daniel Ebner, Davide Danovi, Hiromitsu Nakauchi, and S. Tamir Rashid

Table S1. Details of hepatocyte donors, i-Hep cell lines and negative controls, related to text in Results.

Adult Livers - History of liver disease, Hepatitis B, Hepatitis C and HIV excluded				
Age of Donor	Race	Gender	Cell Viability	Comments
21 years old	Caucasian	Male	90%	Tobacco: Yes<br>Alcohol: No<br>BMI 30
21 years old	Caucasian	Male	88%	Tobacco: Yes<br>Alcohol: 2 units/week<br>BMI 24, Heroin use
29 years old	Caucasian	Male	93%	Tobacco use: No<br>Alcohol: No<br>BMI 25.5
30 years old	African-American	Female	89%	Tobacco: No<br>Alcohol: No<br>BMI 33.2
34 years old	Caucasian	Female	95%	Tobacco use: Yes<br>Alcohol: No<br>BMI 26
39 years old	Caucasian	Male	92%	Tobacco: Yes<br>Alcohol: 2 units/week<br>BMI 21, Heroin use
46 years old	Caucasian	Female	90%	Tobacco: No<br>Alcohol: No<br>BMI 24.0
48 years old	Hispanic	Female	90%	Tobacco: No<br>Alcohol: No<br>BMI 25.4
30 years old	African-American	Male	91%	Tobacco: No<br>Alcohol: No<br>BMI: 20.3
**Paediatric Livers - History of liver disease, Hepatitis B, Hepatitis C and HIV excluded**				
Age of Donor	Race	Gender	Cell Viability	Comments
1 month old	Hispanic	Male	83%	Unknown
14 month old	Caucasian	Female	94%	BMI 20.7
20 months	Caucasian	Female	90%	BMI 21
11 years old	Caucasian	Male	86%	Unknown
**Fetal Livers (Maternal serology negative for Hepatitis B, Hepatitis C and HIV)**				
Age of Donor	Race	Gender	Cell Viability	Comments
19 weeks	Caucasian	Unknown	78%	Termination of pregnancy
21 weeks	Caucasian	Unknown	82%	Termination of pregnancy
20 weeks	Caucasian	Unknown	80%	Termination of pregnancy
17 weeks	Caucasian	Unknown	85%	Termination of pregnancy

Negative Controls			
Sample	Age of Donor	Race, Gender	Comments
Fibroblast 1	51 years old	Caucasian, female	Isolated from skin
Fibroblast 2	44 years old	Caucasian, female	Isolated from skin
Fibroblast 3	40 years old	Caucasian	Isolated from foreskin of adult male
iPS RMA	50years old	Male	Undifferentiated iPS cell line
IPS HDB	Unknown	Unknown	Undifferentiated iPS cell line
DU145	Unknown	Unknown	Prostate Cancer Cell Line

iPS derived Hepatocytes (i-Heps)	
i-Hep 1	Wild-type (Rashid et al JCI 2010)
i-Hep 2	Alpha-1 antitrypsin deficiency (Rashid et al JCI 2010)
i-Hep 3	Wild-type (TkDA Takebe et al Nature 2013)

Table S2. List of ECM / Niche Factors Tested, related to text in Results section.

S/N	Protein / Peptide	Source, Catalogue number
1	Human Collagen-1	Abcam Ab7533
2	Human Collagen-2	Abcam Ab 7534
3	Human Collagen-3	Abcam Ab 7535
4	Human Collagen-4	Abcam Ab7536
5	Human Fibronectin	Merck Millipore FC010
6	Human Laminin 111	Biolamina, Lamscreen KT202
7	Human Laminin 211	Biolamina, Lamscreen KT202
8	Human Laminin 332	Biolamina, Lamscreen KT202
9	Human Laminin 411	Biolamina, Lamscreen KT202
10	Human Laminin 421	Biolamina, Lamscreen KT202
11	Human Laminin 511	Biolamina, Lamscreen KT202
12	Human Laminin 521	Biolamina, Lamscreen KT202
13	Human CYR61	R&D systems, 4055-CR-050
14	Human LRRCC17	Abcam, Ab 160894
15	Human Vitronectin	R&D systems, 2308-VN-050
16	Human Wnt3a	R&D systems, 5036-WN-010
17	Human Wnt5a	R&D systems, 645-WN-010
18	Human Wnt 5b	R&D systems, 7347-WN-025
19	Human Wnt7a	R&D systems, 3008-WN-025
20	Mouse Wnt9a	R&D systems, 8148-WN-025
21	Mouse Wnt9b	Stanford University, Nusse Lab
22	Human EGFL7	Abcam, Ab 218567
23	Human Interleukin 3	R&D systems, 203-IL-010
24	Human Interleukin 4	R&D systems, 6507-IL-010
25	Human Interleukin 6	R&D systems, 7270-IL-025
26	Adrenaline	Sigma Aldrich, Y0000882
27	Serotonin	Sigma Aldrich, H9523
28	Levothyroxine	Sigma Aldrich, L0570000
29	Dexamethasone	Sigma Aldrich, D4902-25MG
30	Insulin	Sigma Aldrich, I3536
31	Ascorbic acid	Sigma Aldrich, PHR1008-2G
32	Protein ORLA 153 (RGDS sequence)	ORLA Proteins, ORLA 153
33	Protein ORLA 164 (PHSRN sequence)	ORLA Proteins ORLA 164
34	Protein ORLA 208 (CS3/Variable domain)	ORLA Proteins ORLA 208
35	Protein ORLA 162 (IKVAV sequence)	ORLA Proteins ORLA 162
36	Protein ORLA 187 (VQLRNGFPYFSY sequence)	ORLA Proteins ORLA 187
37	Protein ORLA 188 (GLLFYMARINHA sequence)	ORLA Proteins ORLA 188
38	Protein ORLA 189 (IKVSV sequence)	ORLA Proteins ORLA 189
39	Protein ORLA 163 (YIGSR sequence)	ORLA Proteins ORLA 163
40	Protein ORLA 165 (MNYYSNS sequence)	ORLA Proteins ORLA 165
41	Protein ORLA 185 (GTPGPQGIAGQRW sequence)	ORLA Proteins ORLA 185
42	Protein ORLA 186 (VEGF mimetic)	ORLA Proteins ORLA 186
43	Protein ORLA 175 (IPKASSVPTELSAISMLYYLDENEKVVLK)	ORLA Proteins ORLA 175
44	Protein ORLA 176 (PQVTRGDVFTM sequence)	ORLA Proteins ORLA 176
45	Protein ORLA 177 (KKQRFRHRNRKGYRSQ sequence)	ORLA Proteins ORLA 177
46	Protein ORLA 178 (VDTYDGRGDSVVYGLRSKSK sequence)	ORLA Proteins ORLA 178
47	Protein ORLA 174 (FHRRIKA Heparin binding)	ORLA Proteins ORLA 174
48	Protein ORLA 179 (VFDNFVLK sequence)	ORLA Proteins ORLA 179
49	Protein ORLA 90 (Fibroblast Growth Factor 1 - acdic)	ORLA Proteins ORLA 90
50	Protein ORLA 128 (Fibroblast Growth Factor 2 - basic)	ORLA Proteins ORLA 128
51	Protein ORLA 181 (Fibroblast Growth Factor 4)	ORLA Proteins ORLA 181
52	Protein ORLA 183 (Stem Cell Factor soluble kit ligand)	ORLA Proteins ORLA 183
53	Protein ORLA 184 (Sonic hedgehog)	ORLA Proteins ORLA 184
54	Protein ORLA 167 (Epidermal Growth Factor)	ORLA Proteins ORLA 167
55	Protein ORLA 180 (Leukaemia Inhibitory Factor)	ORLA Proteins ORLA 180
56	Protein ORLA 190 (GM-Colony Stimulating Factor)	ORLA Proteins ORLA 190
57	Protein ORLA 18 (Protein A)	ORLA Proteins ORLA 18
58	Protein ORLA 85 (Protein G)	ORLA Proteins ORLA 85

Table S3. Primary and secondary antibodies used for immunofluorescence, related to immune-fluorescent staining in Experimental Procedures .

**Primary Antibody [Catalogue Number]**	**Species**	**Supplier of Primary Antibody**	**Secondary Antibody [Catalogue number]**	**Supplier of Secondary Antibody**
Albumin (ALB) [A80-129A]	Goat	Bethyl	Alexa 647 Donkey Anti-Goat [A21447]	Thermo Scientific
A1AT [HM2358]	Mouse	Hycult	Alexa 488 donkey anti mouse [R37114]	Thermo Scientific
Alpha-fetoprotein (AFP) [AB3980]	Mouse	Abcam	Alexa 568 Donkey Anti-Mouse [A10037]	Thermo Scientific
CYP2A6 [Nil]	Hen	Collaborator (University of Eastern Finland)	Alexa 488 Donkey Anti-Hen [703-545-155]	Jackson Immuno-Research Laboratories
HNF4α [AB92378]	Rabbit	Abcam	Alexa 568 Donkey Anti-Rabbit [A10042]	Thermo Scientific

Table S4. Human Taqman probe and primers for RT-PCR analysis were from Thermo Fisher Scientific, related to Real-Time PCR analysis in Experimental Procedures

Target	Cat	Dye
ACTB (Housekeeper)	Hs01060665_g1	VIC
ALBUMIN	Hs00609411_m1	FAM
HNF4A	Hs00230853_m1	FAM
ASGR1	Hs01005019_m1	FAM
CEBP/A	Hs00269972_s1	FAM
CYP1A1	Hs01054797_g1	FAM
CYP2C9	Hs02383631_s1	FAM

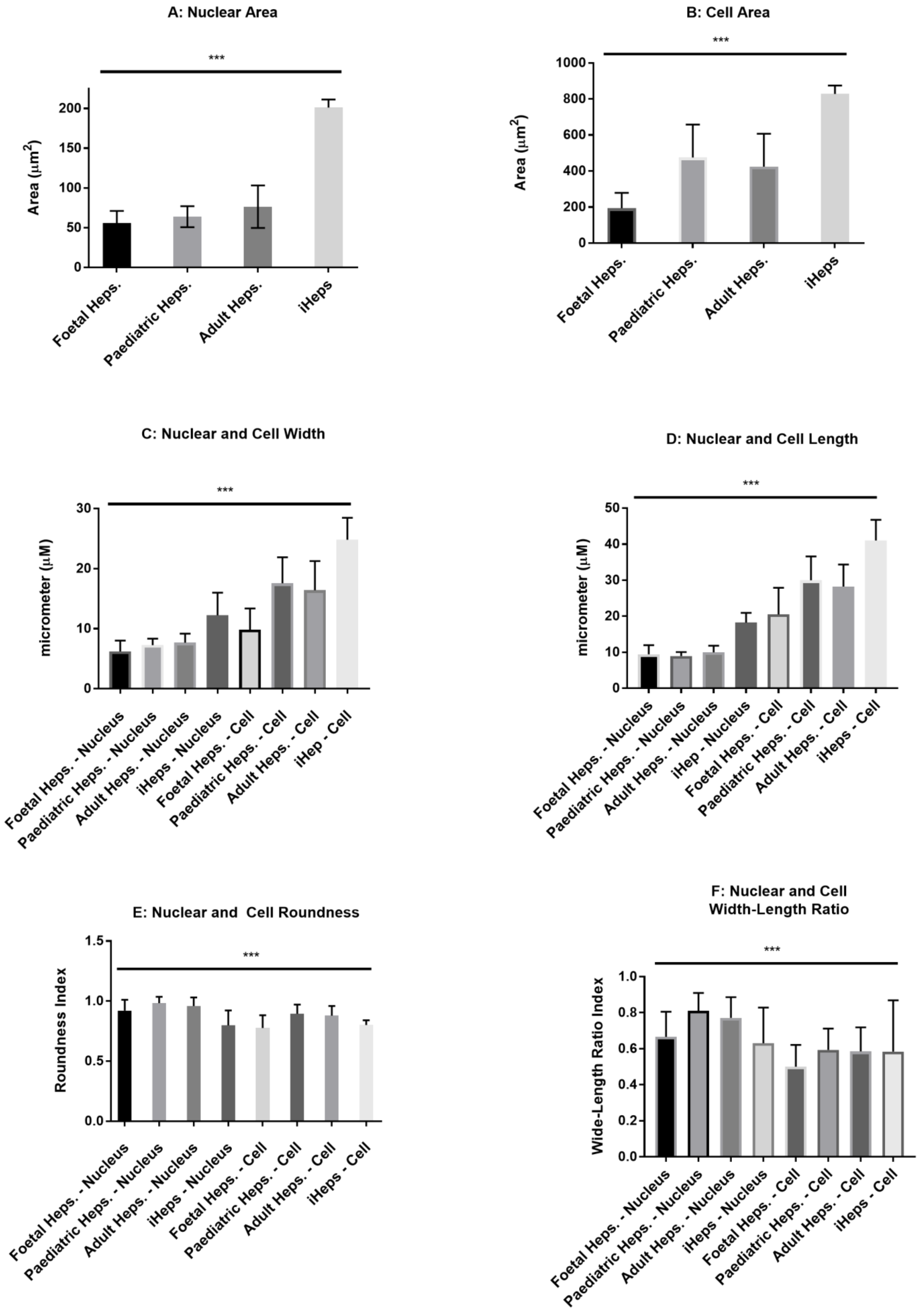

Figure S1. Measured morphological parameters of human hepatocytes by A: nuclear area, B: cell area, C: nuclear and cell width, D: nuclear and cell length, E: nuclear and cell roundness and F: nuclear and cell width-length ratio, related to text in Results section.

(A)

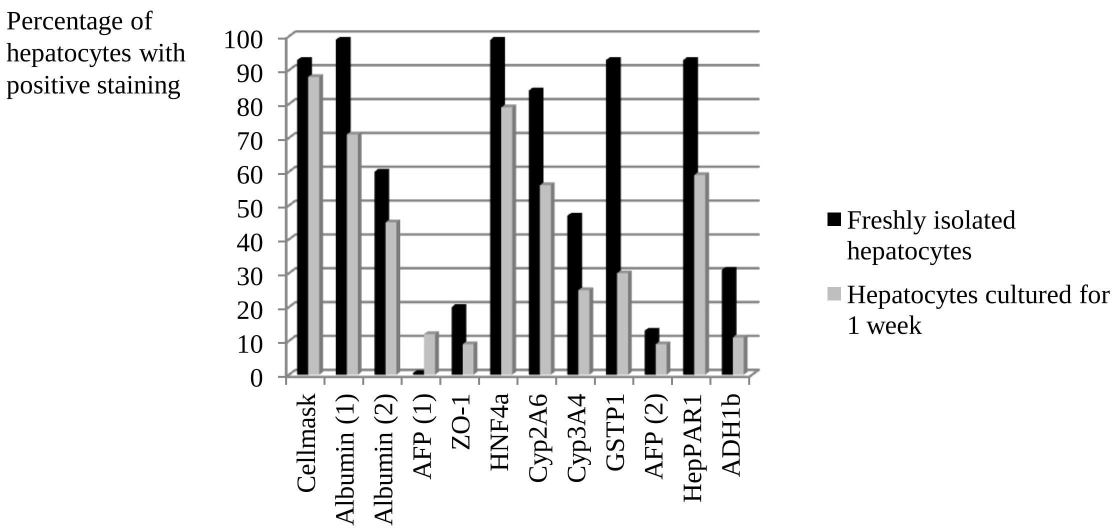

(B)

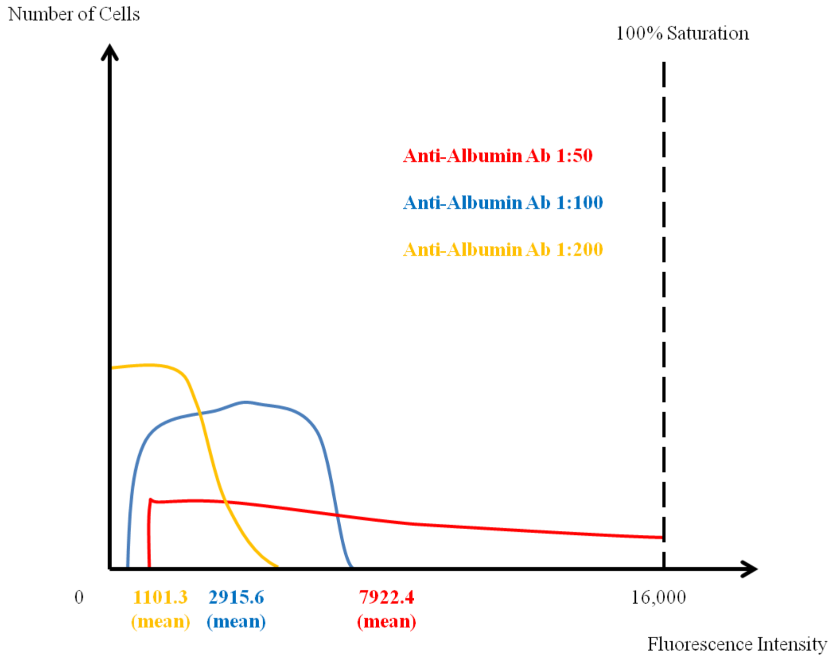

Excitation was fixed at 100% and exposure time was 200ms

Increasing excitation and exposure times stretched the graphs towards the right and caused more cells to approach 100% saturation.

Decreasing excitation and exposure times resulted in a left shift of the graphs towards under-capture of hepatocyte populations.

Figure S2. (A) Performance of various immuno-flourescent antibodies evaluated to screen for hepatocyte likeness (B) High concentrations of Albumin antibody (1:50) resulted in a proportion of cells with 100% saturation. Low concentrations of Albumin antibody (1:200) resulted in under-capture of the cell population with weak staining in a significant proportion of hepatocytes. The optimum concentration of albumin antibody was found to be 1:100. All experiments were performed on 3 separate populations of adult hepatocytes. Figure S2 is related to the text in the Results section.

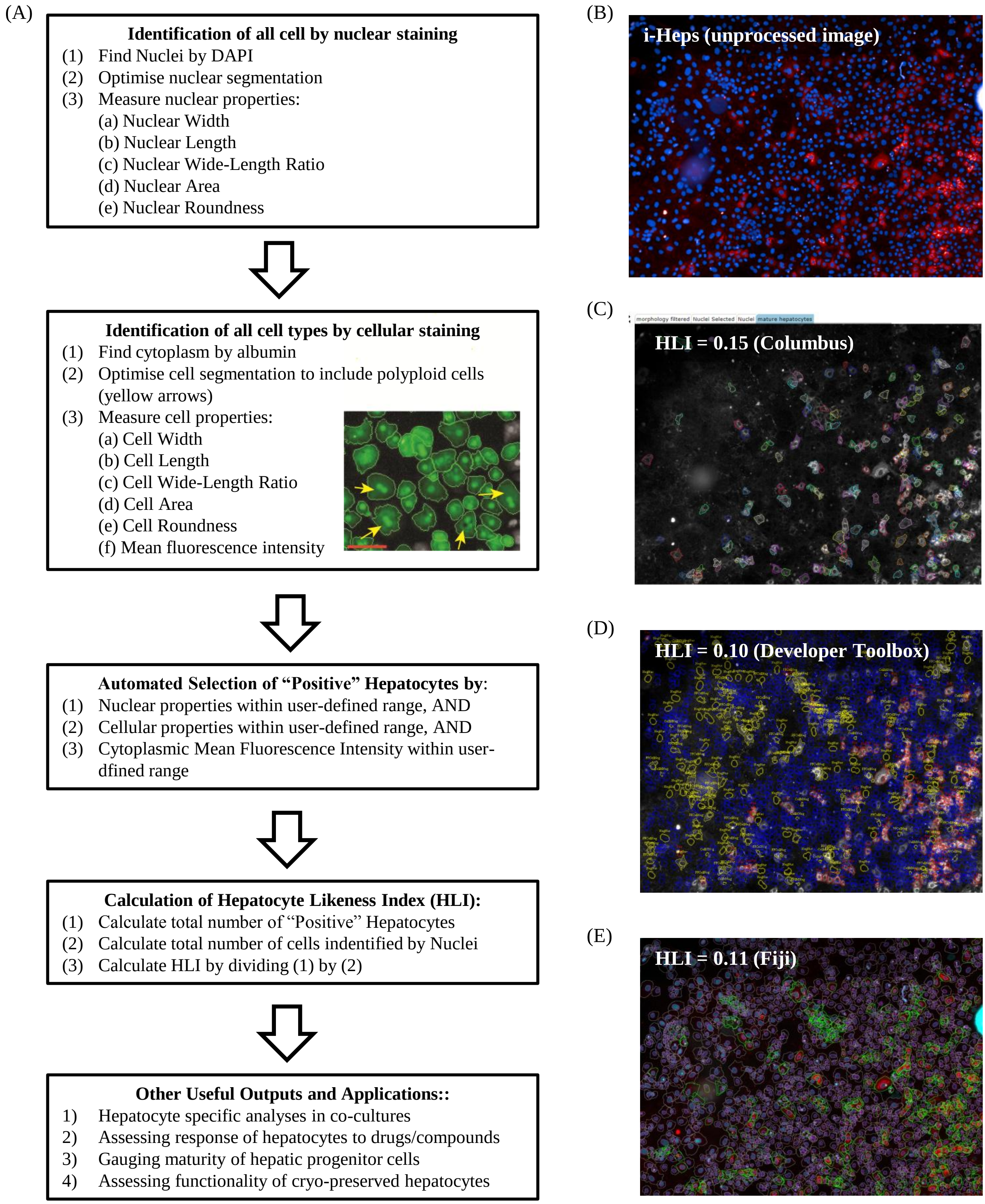

Figure S3. (A) Steps in developing our (Columbus) pipeline using "building blocks" for the derivation of the Hepatocyte Likeness Index; HLI. (B) Unprocessed, reference image of i-Heps that the following pipelines were tested against: (C) Columbus pipeline identifies and highlights mature hepatocytes in grey. (D) Developer Toolbox pipeline identifies mature hepatocytes and highlights these cells in red. (E) ImageJ pipeline identifies mature hepatocytes and highlights these cells in green. (D) and (E) are prototype pipelines created to demonstrate the reproducibility of our Columbus algorithm. (B) to (E) is a selected image from 3 experimental replicates using i-Heps derived from n=3 human iPS cell lines. Pipelines available upon request. Figure S3 is related to text in Results section.

Figure S4. Forward and reverse primers designed for RT-qPCR using PrimerQuest tool (Integrated DNA Technologies), related to Real Time PCR analysis in Experimental Procedures section . The sequences were analyzed using BLAST for specificity for the target sequence at used at 250nM concentration. ALB: albumin, AFP: α-fetoprotein, ACTB: β-actin, FOXA1: forkhead box protein A1, CYP3A4: cytochrome P3A4, HNF4A: hepatocyte nuclear factor 4 alpha,

**Target**	**Forward (5’-3’)**	**Reverse (5’-3’)**
**ALB**	CGTCGAGATGCACACAAGA	GATACTGAGCAAAGGCAATCAAC
**AFP**	TGAATCCAGAACACTGCATAGAA	TATGGTAGCCAGGTCAGCTA
**ACTB**	CTTCCTTCCTGGGCATGG	GTACAGGTCTTTGCGGATGT
**FOXA1**	AGGGCTGGATGGTTGTATTG	GAGTAGGCCTCCTGCGT
**CYP3A4**	CCTGGTGCTCCTCTATCTATATG	AGCCCTTATGGTAGGACAAA
**HNF4A**	ACATGTACTCCTGCAGATTTAG	CCCGGAAGCATTTCTTGA
